# Green-Solvent Processed Blade-Coating Organic Solar Cells with an Efficiency Approaching 19% Enabled by Alkyl-Tailored Acceptors

**DOI:** 10.1007/s40820-023-01208-0

**Published:** 2023-11-02

**Authors:** Hairui Bai, Ruijie Ma, Wenyan Su, Top Archie Dela Peña, Tengfei Li, Lingxiao Tang, Jie Yang, Bin Hu, Yilin Wang, Zhaozhao Bi, Yueling Su, Qi Wei, Qiang Wu, Yuwei Duan, Yuxiang Li, Jiaying Wu, Zicheng Ding, Xunfan Liao, Yinjuan Huang, Chao Gao, Guanghao Lu, Mingjie Li, Weiguo Zhu, Gang Li, Qunping Fan, Wei Ma

**Affiliations:** 1 State Key Laboratory for Mechanical Behavior of Materials, Xi’an Jiaotong University, Xi’an, 710049 People’s Republic of China; 2 Jiangsu Engineering Research Center of Light-Electricity-Heat Energy-Converting Materials and Applications, School of Materials Science and Engineering, Changzhou University, Changzhou, 213164 People’s Republic of China; 3 Key Lab of Fluorine and Silicon for Energy Materials and Chemistry of Ministry of Education/National Engineering Research Center for Carbohydrate Synthesis, Jiangxi Normal University, 99 Ziyang Avenue, Nanchang, 330022 People’s Republic of China; 4 Department of Electronic and Information Engineering, Research Institute for Smart Energy (RISE), The Hong Kong Polytechnic University, Kowloon, 999077 Hong Kong People’s Republic of China; 5 School of Materials Science and Engineering, Xi’an University of Science and Technology, Xi’an, 710054 People’s Republic of China; 6 Department of Applied Physics, The Hong Kong Polytechnic University, Kowloon, 999077 Hong Kong People’s Republic of China; 7 Advanced Materials Thrust, Function Hub, The Hong Kong University of Science and Technology, Nansha, Guangzhou, People’s Republic of China; 8 School of Chemistry and Chemical Engineering, Beijing Institute of Technology, Beijing, 100081 People’s Republic of China; 9 Key Laboratory of Applied Surface and Colloid Chemistry, Ministry of Education, Shaanxi Key Laboratory for Advanced Energy Devices, Shaanxi Engineering Lab for Advanced Energy Technology, School of Materials Science and Engineering, Shaanxi Normal University, Xi’an, 710119 People’s Republic of China; 10 Frontier Institute of Science and Technology, Xi’an Jiaotong University, Xi’an, 710054 People’s Republic of China; 11 Xi’an Key Laboratory of Liquid Crystal and Organic Photovoltaic Materials, State Key Laboratory of Fluorine & Nitrogen Chemicals, Xi’an Modern Chemistry Research Institute, Xi’an, 710065 People’s Republic of China

**Keywords:** Alkyl-tailored guest acceptors, Blade-coating, Green solvent processing, Stability, Organic solar cells

## Abstract

Power-conversion-efficiencies (PCEs) of organic solar cells (OSCs) in laboratory, normally processed by spin-coating technology with toxic halogenated solvents, have reached over 19%. However, there is usually a marked PCE drop when the blade-coating and/or green-solvents toward large-scale printing are used instead, which hampers the practical development of OSCs. Here, a new series of *N*-alkyl-tailored small molecule acceptors named YR-SeNF with a same molecular main backbone are developed by combining selenium-fused central-core and naphthalene-fused end-group. Thanks to the *N*-alkyl engineering, NIR-absorbing YR-SeNF series show different crystallinity, packing patterns, and miscibility with polymeric donor. The studies exhibit that the molecular packing, crystallinity, and vertical distribution of active layer morphologies are well optimized by introducing newly designed guest acceptor associated with tailored *N*-alkyl chains, providing the improved charge transfer dynamics and stability for the PM6:L8-BO:YR-SeNF-based OSCs. As a result, a record-high PCE approaching 19% is achieved in the blade-coating OSCs fabricated from a green-solvent *o*-xylene with high-boiling point. Notably, ternary OSCs offer robust operating stability under maximum-power-point tracking and well-keep > 80% of the initial PCEs for even over 400 h. Our alkyl-tailored guest acceptor strategy provides a unique approach to develop green-solvent and blade-coating processed high-efficiency and operating stable OSCs, which paves a way for industrial development.

**Supplementary Information:**

The online version contains supplementary material available at 10.1007/s40820-023-01208-0.

## Highlights

- Alkyl-tailored Y-SMAs named YR-SeNF series with near-infrared absorption, different molecular crystallinity and self-assembly abilities are developed.
- The related organic solar cells (OSCs) with an active layer processed from halogen-free solvents and spin-coating-free technologies achieve a ~ 19% efficiency.
- Ternary OSCs offer a robust operating stability under MPP tracking and well-keep > 80% of the initial efficiency for even over 400 h.

**Supplementary Information:**

The online version contains supplementary material available at 10.1007/s40820-023-01208-0.

## Graphical abstract

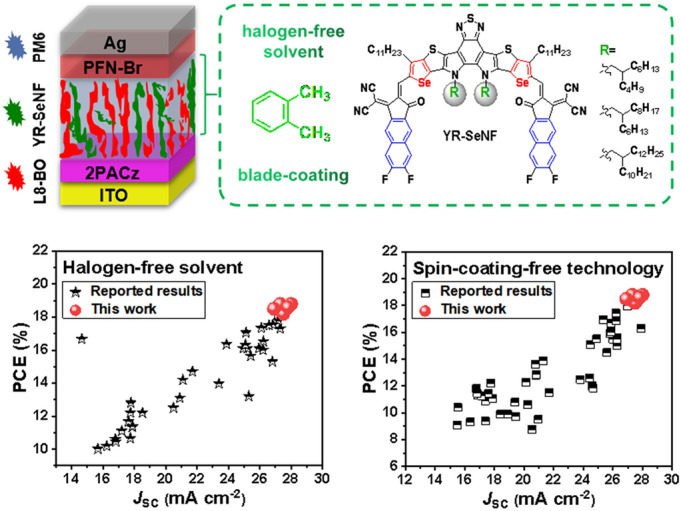

## Introduction

Organic solar cells (OSCs) have attracted great attention due to the advantages of low-cost, versatile applications in flexible electronics, and potential large-area printing processability [1–6]. Mainly due to the remarkable breakthrough of non-fullerene small molecule acceptors (SMAs) recently, especially superstar Y-series SMAs (Y-SMAs) with a donor–acceptor-donor (DAD)-fused central-core and two benzene-fused electron-deficient end-groups, OSCs have achieved impressively high-power conversion efficiencies (PCEs) of over 19%, which greatly lights up the commercial prospect of OSCs [7–15]. However, these high PCEs are usually obtained from spin-coating process and low-boiling-point toxic halogenated solvents such as chloroform, while the former limits the large-area fabrication of OSCs, and the latter will cause serious environmental pollution [16, 17]. When turning to the blade-coating process toward large-scale printing and high-boiling-point eco-friendly solvents required for industrial development, there is usually a marked PCE drop.

The PCE drop of OSCs processed from halogen-free high-boiling point solvents is mainly due to the poor solubility of photovoltaic materials, such as Y6 with short sized alkyl chains shows low solubility in *o*-xylene, leading to strong self-aggregation thus serious phase separation in active layers [18, 19]. Previous researches have attempted to carry out alkyl chain engineering on Y-SMAs to solve the solubility challenge, so as to be able to use halogen-free high-boiling-point solvents to fabricate efficient OSCs [20–26]. However, while improving the solubility, long-sized alkyl chains usually break the tight packing of backbone, which leads to weak molecular ordered assembly and poor charge transfer properties [27–32]. On the other hand, the PCE drop of OSCs processed with blade-coating technology is mainly due to the difficulty in precise control of the crystallization and phase separation kinetics during film formation, commonly resulting in undesirable active layer morphology [33–39]. As a result, although great efforts have been made to alter the alkyl chains of Y-SMAs and optimize the film-formation process, the success in achieving high-efficiency OSCs processed with eco-friendly solvents and blade-coating technology so far is still constrained.

**Fig. 1:**
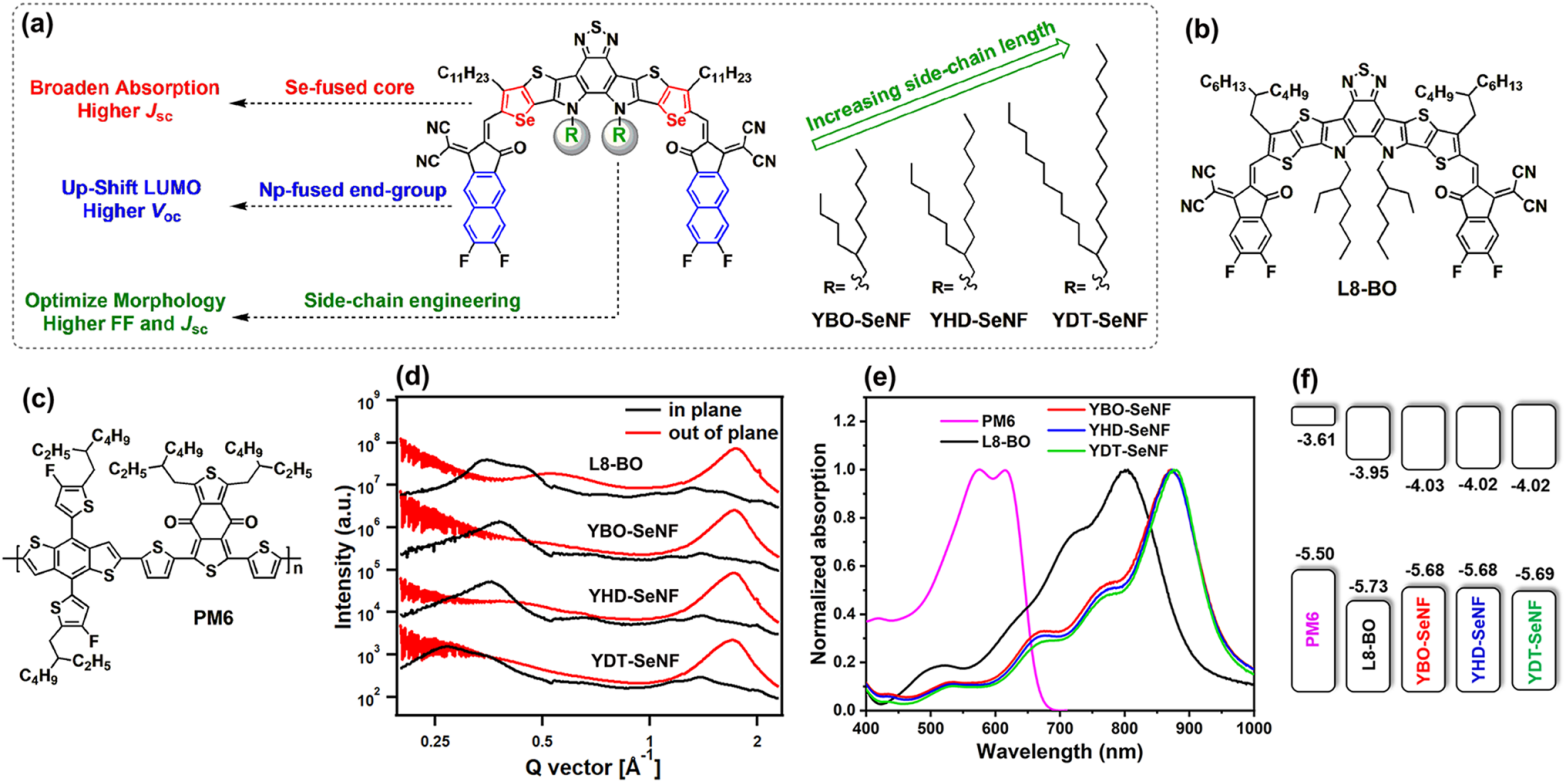
**a** Molecular structures and design strategy of YR-SeNF series as guest acceptors used in the PM6:L8-BO host system. **b, c** Molecular structures of PM6 and L8-BO. **d** Line-cuts from GIWAXS profiles of the L8-BO and YR-SeNF series in neat films. **e** Absorption spectra and **f** energy levels of PM6, L8-BO, and YR-SeNF series

Here, a series of newly designed *N*-alkyl-tailored Y-SMAs (named YR-SeNF, including YBO-SeNF, YHD-SeNF, and YDT-SeNF) are developed by combining selenophene (Se)-fused central-core with naphthalene (Np)-fused end-groups (Fig. 1a), which show near-infrared (NIR)-absorption, different molecular crystallinity and self-assembly abilities. The studies exhibit that these *N*-alkyl-tailored YR-SeNF as the key guest components in binary PM6:L8-BO host system can well manipulate morphologies of blade-coating active layers processed with halogen-free and high-boiling-point *o*-xylene solvent, such as molecular packing, crystallinity, and vertical distribution, resulting in the improved charge transfer dynamics and stability for OSCs. Benefiting from the above advantages, we successfully achieve a record-high PCE approaching 19% in the blade-coating OSCs processed from *o*-xylene solvent. Notably, due to the outstanding compatibility between guest YHD-SeNF and host L8-BO, ternary OSCs offer robust operating stability under maximum-power-point (MPP) tracking and well-keep > 80% of the initial PCEs for even over 400 h.

## Experimental Section

### Device Fabrication and Characterization

The OSCs with a device structure of ITO/2PACz/active layer/PFN−Br/Ag were fabricated under conditions as follows. ITO substrates were first scrubbed, dried overnight in an oven, and then treated by UV−Ozone for 30 min before use. The 2PACz was dissolved in ethanol with a concentration of 0.5 mg mL^−1^. ITO substrates were then dipped into 2PACz solution with a temperature of 80 °C for 10 min, then taken out and washed by pure ethanol. PM6:L8−BO:YR−SeNF (1:1.2:0, 1:1:0.2, 1:0.8:0.4, and 1:0:1.2) blends were dissolved in *o*−xylene with 1,4−diiodobenzene (DIB) as solid additive (100% in w/w) under 40 °C for 3 h to mix intensively in a N_2_−filled glove box, in which the D:A solid concentration is 25 mg mL^−1^ in total [40]. The blend solutions were blade−coated immediately on the top of 2PACz layer with a speed of 30 mm s^−1^ followed by a 100 °C thermal annealing. The optimal active layer thickness measured by a Bruker dektak XT stylus profilometer was about 100 nm. Then, a thin PFN−Br layer (0.5 mg mL^−1^ in methanol with 0.25 wt% melamine) was blade−coated (10 mm s^−1^ moving speed) on the active layer, followed by the deposition of Ag (evaporated under 5×10^−4^ Pa through a shadow mask). The current density–voltage (*J−V*) curves of OSCs were measured using a keysight B2901A source meter in glove box under AM 1.5G (100 mW cm^−2^) using an Enlitech solar simulator. The device contact area was 0.042 cm^2^, device illuminated area during testing was 0.0324 cm^2^, which was determined by a mask. The external quantum efficiency (EQE) spectra were measured using a solar cell spectral response measurement system QE−R3011 (Enlitech). The light intensity at each wavelength was calibrated using a standard monocrystalline Si photovoltaic cell. The MPP tracking was carried out upon epoxy encapsulated devices under 1−sun white LED array in air. The whole tracking condition (temperature and humidity) is co−controlled by air−conditioner and blowing cooling setups.

## Results and Discussion

### Photophysical and Electrochemical Properties

Design strategy and synthetic routes of YR-SeNF series with different *N*-alkyl substitutions (YBO-SeNF with 2-butyloctyl, YHD-SeNF with 2-hexyldecyl, and YDT-SeNF with 2-decyltetradecyl) are presented in Fig. 1a and S1. The characterizations of molecular structure (including^1^H/^13^C NMR and mass spectra) for YR-SeNF series are summarized in Figs. S2-S12, respectively. The gradually extended *N*-alkyl chain length in YR-SeNF series offers better solubility in halogen-free and high-boiling-point solvents such as *o*-xylene. In the grazing incidence wide-angle X-ray scattering (GIWAXS) measurements (Fig. 1d, S13, and Table S1) of the blade-coating neat films, compared to YDT-SeNF with the longest *N*-alkyl chains has a diffused (100) peak in in-plane (IP) direction, both of YBO-SeNF and YHD-SeNF achieve the sharp (100) peaks. With the *N*-alkyl sized extension, YR-SeNF series show gradually increased π-π stacking distances of 3.659–3.726 Å and decreased crystal coherence lengths (CCLs) of 15.01–16.57 Å, indicating that *N*-alkyl chain engineering successfully regulates the molecular crystallinity and ordered stacking. These properties offer some information in controlling crystallization and morphology of blade-coating active layers, especially when introducing YR-SeNF series as the third component into PM6:L8-BO host system, which well be elaborated in the following sections.

In both dilute *o*-xylene solutions (Fig. S14) and neat films (Fig. 1e), YR-SeNF series show nearly identical absorption spectra. As shown in Fig. S15 of molecular temperature dependent absorption in dilute *o*-xylene solution, benefiting from the strong intermolecular interaction caused by the combination of Se-fused central-core and Np-fused end-groups, all of YR-SeNF series only show slightly decreased absorption intensity as the temperature increases from 30–100 °C [41]. YR-SeNF series exhibit much sharper 0–0 peaks which belong to *J*-aggregation compared to L8-BO in films, indicating more orderly intermolecular π-π stacking. Thanks to that selenium atom has a looser delocalized electron cloud than sulfur one to form better orbital overlap and stronger quinoidal character in π-conjugated system [42, 43], YR-SeNF series display a 75 nm red-shifted absorption peak, 65 nm extended absorption onset, and much smaller optical bandgap (*E*_g_^opt^) of 1.27 eV compared to L8-BO (1.36 eV), which is more complementary to PM6 and well-fill in the photon capture deficiency of L8-BO in the NIR region of > 900 nm. As shown in Fig. S16, all of L8-BO:YR-SeNF (0.8:0.4) binary blends with a small amount of YR-SeNF as guest component show obviously red-shifted absorption onsets compared to L8-BO, while they only show slightly blue-shifted absorption spectra compared to YR-SeNF, suggesting that each component kept its own photon capturing behavior to large extent. A similar phenomenon has been observed in ternary PM6:L8-BO:YR-SeNF blends with different component ratios (Fig. S17). As shown in Fig. S18, L8-BO:YR-SeNF binary acceptors have almost same absorption, suggesting a similar compatibility among them. Differently, ternary PM6:L8-BO:YR-SeNF blends show obviously diverse absorption onsets due to different miscibility between PM6 and YR-SeNF series.

In the contact angle measurements (Fig. S19), the surface free energy (γ) values of PM6, L8-BO, YBO-SeNF, YHD-SeNF, and YDT-SeNF were calculated as 33.45, 39.32, 37.02, 36.06, and 34.15 mN m^−1^, respectively. The related interaction parameters (χ) between PM6 and YR-SeNF series were estimated as 0.090, 0.049, and 0.001 in the above order by the Flory–Huggins method, while the χ between L8-BO and YR-SeNF series were estimated as 0.035, 0.071, and 0.180, respectively [44, 45]. The almost imperceptible interaction between PM6 and YDT-SeNF indicates good miscibility between them, while YBO-SeNF and YHD-SeNF exhibit a much stronger interaction with PM6 and less miscibility.

Cyclic voltammograms (CV) were performed to study the molecular energy levels of YR-SeNF series. As shown in Fig. 1f and S20, YR-SeNF series with extended *N*-alkyl chains show similar lowest unoccupied molecular orbital (LUMO) energy levels, which are consistent with their nearly identical absorption spectra. Moreover, compared to L8-BO host [46], YR-SeNF guests have decent LUMO levels (Δ*E*_LUMO_ =  ~ 0.07 eV), even the latter ones have obviously red-shifted absorption onset of 65 nm, which is expected to achieve both high open-circuit voltage (*V*_OC_) and short-circuit current density (*J*_SC_).

### Molecular Dynamics Simulations

**Fig. 2:**
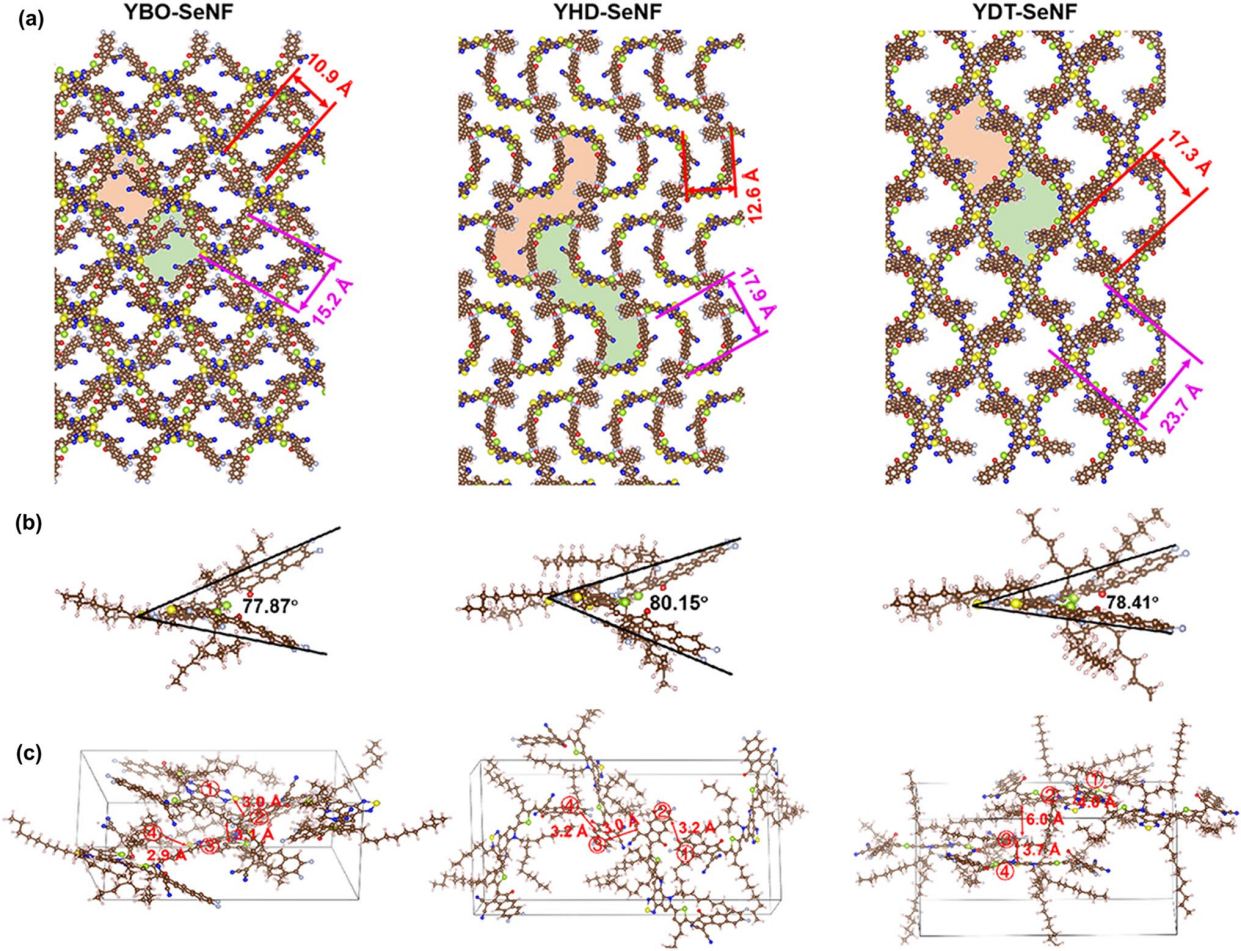
**a** Packing patterns of YR-SeNF series from MD simulation, where N-alkyl chains are omitted for clarity. **b** Geometries of YR-SeNF series. **c** Tetramer configuration cells of YR-SeNF series, where the numbers in Figures represent the closest distance between adjacent molecular backbones within a cell

Molecular dynamics (MD) simulations were carried out to understand the intermolecular packing of alkyl-tailored YR-SeNF series [47, 48]. As depicted in Fig. 2a, YR-SeNF series with different *N*-alkyl substitutions have dramatically different packing patterns. Among them, YBO-SeNF and YDT-SeNF show similar rectangle shaped voids with different size, while YHD-SeNF plays a unique role in constructing three-dimensional (3D) network with special *S*-shaped voids [49]. Unique *S*-shaped voids in YHD-SeNF indicate that molecular packing pattern not only derives from the direct influence of side-chain steric effect but also the indirect influence of side-chain by changing the geometric shape of backbone. With the extension of *N*-alkyl in YR-SeNF series, the voids increase by ~ 1.6 times from 10.9 × 15.2 to 17.3 × 23.7 Å^2^ due to the steric hindrance of *N*-alkyl chains, which will affect their solution processability especially in halogen-free solvents with high-boiling point. Moreover, the geometries of YR-SeNF series need to be attention due to *N*-alkyl chains also can effect molecular backbone, which is related to intermolecular aggregation and blend morphology. As illustrated in Fig. 2b, YR-SeNF series show a similar banana curved and helical molecular geometry. Molecular angles built from the two F atoms in end-groups and S atom in center (∠FSF) are 77.81° for YBO-SeNF, 80.15° for YHD-SeNF, and 78.41° for YDT-SeNF, respectively. YHD-SeNF has more twisted backbone with a bigger angle due to its larger *N*-alkyl chains compared to YBO-SeNF. YHD-SeNF shows a larger torsion angle compared to YDT-SeNF, mainly due to the middle *N*-alkyl chains in YHD-SeNF can be wrapped within the molecule. In the case of YDT-SeNF, *N*-alkyl chains are too large to wrap within its molecule and are squeezed onto both the sides of molecular backbone, resulting in a smaller intramolecular steric effect and thus, a smaller molecular twist. The above results partially explain why YR-SeNF acceptors have distinctive packing patterns. As shown in Fig. S21 and Table S2, using the space-charge-limited-current (SCLC) method, YBO-SeNF and YDT-SeNF have slightly higher electron-mobilities (*μ*_e_) of ~ 1.9 × 10^−4^ cm^2^ V^−1^ s^−1^ compared to YHD-SeNF (1.65 × 10^−4^ cm^2^ V^−1^ s^−1^), which are also related to their twisted backbone (Fig. 2b).

The packing distance differences of YR-SeNF series are further looked into (Fig. 2c), which more reflects the intermolecular steric hindrance of *N*-alkyl chains. YBO-SeNF with a relatively small steric hindrance can be together closer (2.9 Å) and thus, may cause excessive aggregation. YDT-SeNF with long-sized *N*-alkyl chains has far away packing from each other, even up to 6 Å. The middle *N*-alkyl chains of YHD-SeNF result in a suitable steric hindrance that allows the intermolecular stacking distance to be 3.0–3.2 Å, which does not cause excessive aggregation, nor will it be too far apart to facilitate charge transport. The above results indicate that suitable *N*-alkyl chains may balance intramolecular and intermolecular steric hindrance and thus, form unique stacking structures for improving charge transfer and charge transport dynamics in active layers [50, 51].

### Photovoltaic Properties and Stability

**Fig. 3:**
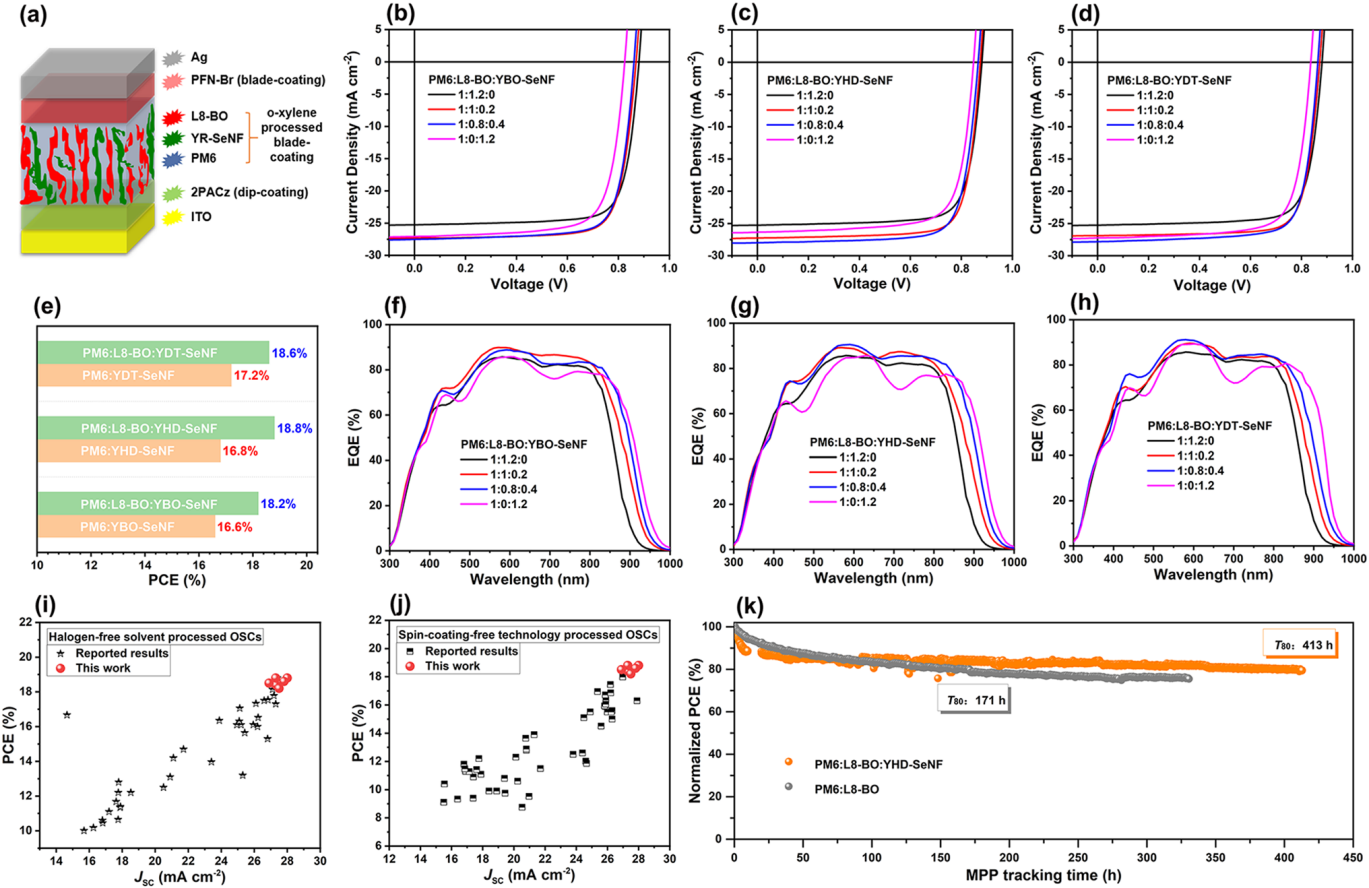
**a** Device structure of the spin-coating-free OSCs in this work. **b–d** *J-V* plots of OSCs under the illumination of AM 1.5G, 100 mW cm^2^. **e** Comparison of PCEs in column chart form between the YR-SeNF-based binary and ternary OSCs. **f–h** EQE spectra of OSCs. Statistical PCE vs *J*_SC_ of the OSCs based on active layers processed from **i** halogen-free solvents and **j** spin-coating-free technologies. **k** MPP tracking for the OSCs based on PM6:YHD-SeNF and PM6:L8-BO:YHD-SeNF under a solar simulator with 1-sun intensity

The photovoltaic performances of the OSCs based on binary PM6:YR-SeNF and ternary PM6:L8-BO:YR-SeNF with a device structure of ITO/2PACz/active layer/PFN-Br/Ag were studied by a combining strategy toward industrial development of spin-coating-free (dip-coating for 2PACz layer, blade-coating for active layer and PFN-Br layer) technology and halogen-free high-boiling-point *o*-xylene solvent processing (Fig. 3a). As shown in Fig. 3b–d of the *J-V* curves and Table 1 of photovoltaic parameters, with the extension of *N*-alkyl size in YR-SeNF series, the related OSCs obtained gradually improved PCEs from 16.1 to 17.2%. The PM6:YR-SeNF-based OSCs achieved both lower *V*_OC_ of 0.824–0.848 V and fill factor (FF) of 0.745–0.754 but significantly higher *J*_SC_ of 26.4–27.2 mA cm^−2^ compared to the PM6:L8-BO-based ones (*V*_OC_ = 0.880 V, *J*_SC_ = 25.3 mA cm^−2^, and FF = 0.779). As we expected, the PM6:YR-SeNF-based OSCs obtained higher *V*_OC_ × *J*_SC_ products as 22.33–22.74 compared to the PM6:L8-BO-based one (22.26), implying the effectiveness of our strategy to develop NIR-absorbing Y-SMAs for boosting both photo-current and photo-voltage outputs. All these characteristics, such as relatively narrow absorption of < 900 nm, high *V*_OC_ and FF, suggesting that binary PM6:L8-BO is an ideal candidate to further improve PCE by introducing YR-SeNF series as guest components with high *J*_SC_ due to the broadened absorptions > 975 nm.

**Table 1:** Photovoltaic parameters of the OSCs based on PM6:L8-BO:YR-SeNF with different weight ratios

		*V*_OC_ [V]	*J*_SC_ [mA cm^−2^]^[a]^	FF	PCE [%]
PM6:L8-BO: YBO-SeNF	1:1.2:0	0.880	25.3 (24.7)	0.779	17.3
	1:1:0.2	0.869	27.3 (26.9)	0.776	18.4
	1:0.8:0.4	0.861	27.5 (27.1)	0.772	18.2
	1:0:1.2	0.824	27.1 (26.2)	0.745	16.6
PM6:L8-BO: YHD-SeNF	1:1:0.2	0.874	27.3 (26.7)	0.789	18.8
	1:0.8:0.4	0.866	28.0 (27.6)	0.775	18.8
	1:0:1.2	0.848	26.4 (25.8)	0.753	16.8
PM6:L8-BO: YDT-SeNF	1:1:0.2	0.871	26.9 (26.4)	0.788	18.5
	1:0.8:0.4	0.864	27.8 (27.4)	0.775	18.6
	1:0:1.2	0.836	27.2 (26.7)	0.754	17.2

^a^The integrated *J*_SC_ values were calculated from the EQE spectra

To study the effects of introducing guest YR-SeNF series into binary PM6:L8-BO host system on photovoltaic performance, the PM6:L8-BO:YR-SeNF-based ternary OSCs were fabricated. Surprisingly, all ternary OSCs with different PM6:L8-BO:YR-SeNF ratios (1:1:0.2 and 1:0.8:0.4) achieved much superior device performance compared to their parental binary OSCs. For example, ternary OSCs obtained much higher *J*_SC_ of 27–28 mA cm^−2^, well-kept and even higher FF of 0.772–0.789, and only slightly reduced *V*_OC_ of 0.861–0.874 V compared to the PM6:L8-BO-based OSCs. Moreover, ternary OSCs achieved furtherly improved products of *V*_OC_ × *J*_SC_ (23.43–24.25) compared to their parental binary ones (22.26–22.74), while the YHD-SeNF-based ternary OSCs achieved the highest value. As a result, these ternary OSCs offered an impressive PCE of 18.2%-18.8%, which is much higher than their parental binary devices (~ 17%) (Fig. 2e). Notably, the PCE of 18.8% from the YHD-SeNF-based ternary devices is the highest value among the OSCs based on active layers processed from halogen-free solvents (Fig. 2i and Table S3) and spin-coating-free technologies (Fig. 2j and Table S4). Statistical photovoltaic data from 20 independent OSCs with different component ratios were summarized in Table S5-S14.

As shown in Fig. 2f–h, the EQE measurements were carried out to verify the reliability of *J*_SC_ values in OSCs. With the increase in YR-SeNF content in active layers, the OSCs show gradually broadened EQE spectra in NIR direction. The ternary OSCs based on PM6:L8-BO:YR-SeNF (1:0.8:0.4) with a small amount of YR-SeNF show significantly red-shifted response compared to the PM6:L8-BO-based ones but only slightly blue-shifted response in comparison with the PM6:YR-SeNF-based ones, may be due to good compatibility between L8-BO and YR-SeNF in which each component well-kept own photon absorption behavior (Figs. S16-S17). Moreover, ternary OSCs displayed higher EQE values from 400 to 900 nm compared to binary parental OSCs. Especially, ternary OSCs based on PM6:L8-BO:YR-SeNF (1:0.8:0.4) achieved much higher integrated *J*_SC_ (27.1–27.6 mA cm^−2^) than these of binary OSCs based on PM6:L8-BO (24.7 mA cm^−2^) and PM6:YR-SeNF (25.8–26.7 mA cm^−2^), which are well consistent with the results from *J-V* curves.

As displayed in Fig. 2k, the operational stability of OSCs at MPP tracking was probed under a continuous illumination of 1-sun solar simulator to evaluate their practical application potential [52, 53]. The device based on ternary PM6:L8-BO:YHD-SeNF (1:0.8:0.4) displayed excellent stability and kept > 80% of initial PCE after the MPP tracking for even over 400 h, which is much better than its parental OSCs based on binary PM6:L8-BO.

### Crystallinity, Morphological Properties, and Vertical Phase Distribution

As displayed in Fig. S22 and Table S15, the molecular crystallinity, orientation, and packing properties of binary L8-BO:YR-SeNF (1:0.2 and 0.8:0.4) were performed by using GIWAXS measurements [54–56]. With the extension of *N*-alkyl size in YR-SeNF series, binary L8-BO:YR-SeNF achieve gradually increased π-π stacking distances in out-of-plane (OOP) direction, which is consistent with the YR-SeNF films. In IP direction, binary L8-BO:YR-SeNF shows two independent (100) diffraction peaks. With the increase in YR-SeNF content, these blends display the weakened (100) diffraction peaks of L8-BO but well-keep the ones from YR-SeNF. The above results suggest that YR-SeNF series are good compatible with L8-BO, where each component has its own aggregation behavior, and thus, morphology can be optimized by inserting YR-SeNF series as guest acceptors.

**Fig. 4:**
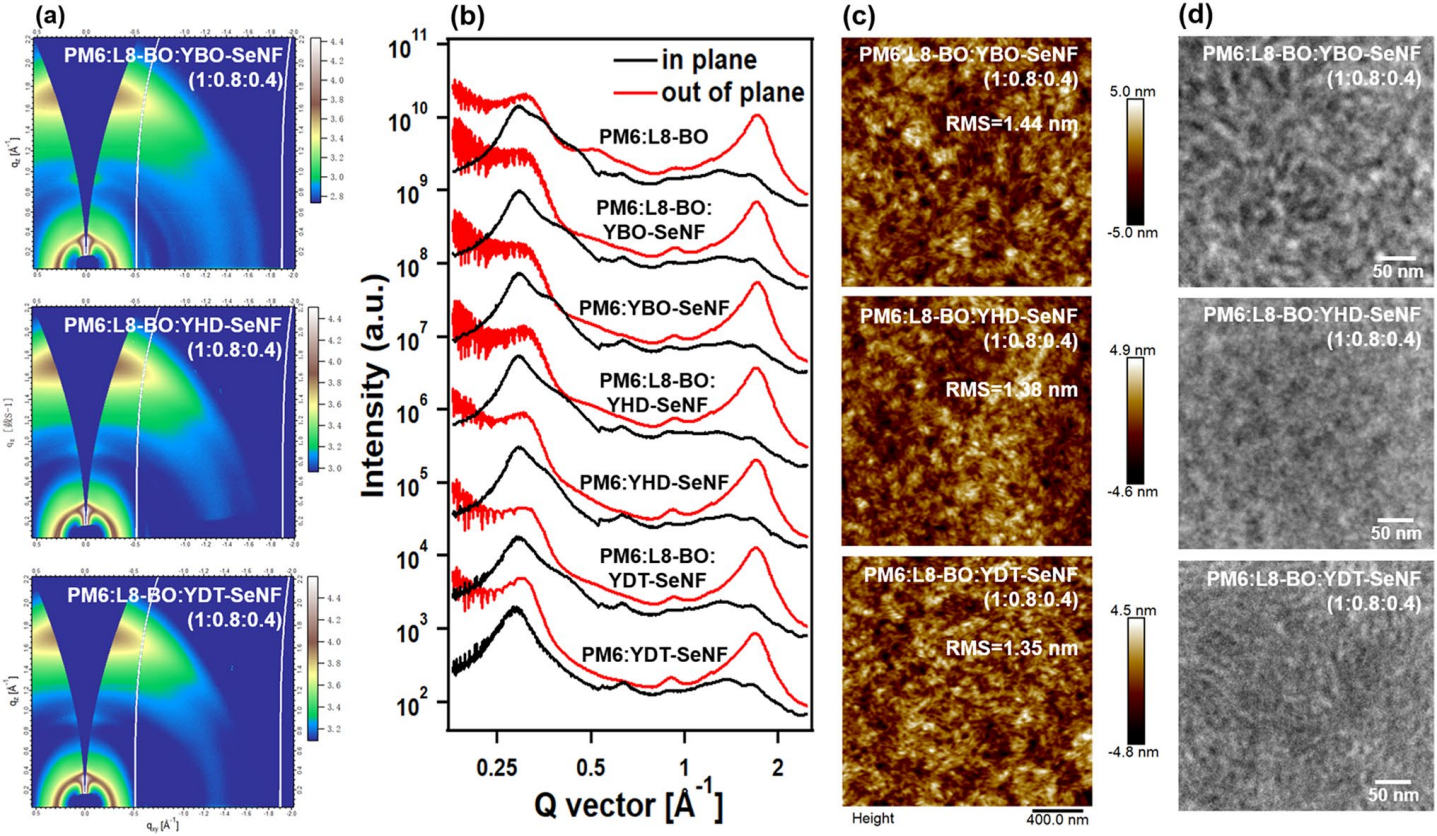
**a** GIWAXS profiles, **c** AFM height images, and **d** TEM images of ternary PM6:L8-BO:YR-SeNF (1:0.8:0.4), respectively. **b** Line-cuts from GIWAXS profiles of PM6:L8-BO, PM6:YR-SeNF, and PM6:L8-BO:YR-SeNF (1:0.8:0.4)

As depicted in Fig. 4a-b, S23-S24, and Table S16, to probe the effects of introducing guest YR-SeNF series into PM6:L8-BO host system on device performance, the blend morphologies of PM6:L8-BO:YR-SeNF with different component ratios were studied. With the extension of *N*-alkyl size in YR-SeNF series, PM6:YR-SeNF binary blends show gradually increased π-π stacking distances and decreased CCLs in OOP direction, which is consistent with the results from YR-SeNF films (Fig. 1d) and MD simulations (Fig. 2c). Similar phenomena have also been observed in ternary PM6:L8-BO:YR-SeNF (1:0.8:0.4). Compared to binary PM6:YBO-SeNF and PM6:YHD-SeNF with two (100) diffraction peaks in IP direction that belong to PM6 at ~ 0.29 Å^−1^ and YR-SeNF at ~ 0.35 Å^−1^, respectively, binary PM6:YDT-SeNF only shows a sharp (100) diffraction peak due to good miscibility between them (Fig. S19). Moreover, ternary PM6:L8-BO:YR-SeNF exhibited an additional (100) diffraction peak at ~ 0.40 Å^−1^ that belongs to L8-BO in comparison with binary PM6:YR-SeNF, which helps to provide more charge transport channels.

Atomic force microscopy (AFM) and transmission electron microscopy (TEM) measurements were carried out to study surface and bulk morphologies of blend films. As shown in Fig. S25, with the *N*-alkyl sized extension of YR-SeNF series, binary PM6:YR-SeNF blends show gradually decreased root-mean-square (RMS) roughness and reduced fiber size, which is consistent with their improved miscibility between them (Fig. S19). Ternary PM6:L8-BO:YR-SeNF blends show decreased RMS roughness as the content of YR-SeNF increases (Figs. S26-S28), which may be due to the fact that the RMS roughness of PM6:L8-BO (2.47 nm) is greater than that of PM6:YR-SeNF (~ 1.3 nm). As shown in Fig. 4c, ternary PM6:L8-BO:YR-SeNF (1:0.8:0.4) blends with uniform fiber structure have similar but gradually decreased RMS roughness from 1.44 to 1.35 nm as the *N*-alkyl sized extension of YR-SeNF series. In the TEM images (Fig. 4d), with the *N*-alkyl sized extension of YR-SeNF series, ternary PM6:L8-BO:YR-SeNF (1:0.8:0.4) blends also show gradually reduced phase separation in accordance with expectation.

**Fig. 5:**
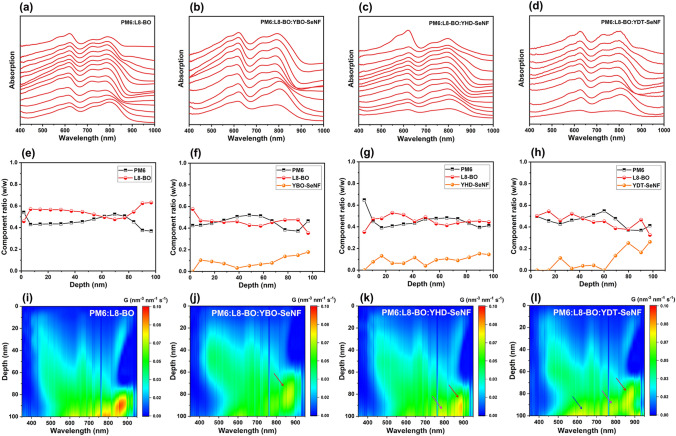
**a–d** Film-depth-dependent light absorption spectra, **e–h** composition distribution, and **i–l** exciton generation contour as a function of film depth for PM6:L8-BO and PM6:L8-BO:YR-SeNF (1:0.8:0.4) blend films, where depth 0 and 100 nm represent the active layer/PFN-Br and active layer/2PACz interfaces, respectively. For exciton generation contour, the incident light is from the ITO/2PACz side

Film-depth-dependent light absorption spectrometry (FLAS) was performed to study the effects of inserting guest YR-SeNF series on vertical phase distribution between donor and acceptor components [57]. As shown in Fig. 5a-d, all of blends show the same absorption peak positions of PM6 at ~ 620 nm throughout whole film, implying the similar molecular packing at different depths, which may help to minimize energetic disorder. By fitting the FLAS curves with the individual absorption of PM6, L8-BO, and YR-SeNF neat films, the composition ratios of active layer materials at different film-depths can be extracted (Fig. 5e-h). PM6:L8-BO:YR-SeNF ternary blends show more balanced vertical phase distribution between PM6 and L8-BO throughout the entire active layers compared to PM6:L8-BO one. Among these ternary blends, PM6:L8-BO:YHD-SeNF displays more uniform vertical phase distribution of guest acceptor, which may help to form smoother energy level gradient in OSCs and thus reduce *V*_OC_ loss (Fig. 3b-d). As displayed in Fig. 5i-l, the exciton generation contour of OSCs was numerically simulated by combining the above FLAS information with an optical transfer matrix model. Compared to YBO-SeNF-based ternary blend, both ternary blends based on YHD-SeNF and YDT-SeNF yielded higher number of excitons from acceptors phase (800 nm), which partly explains their higher *J*_SC_ values in OSCs.

### Charge Transfer and Exciton Lifetimes

**Fig. 6:**
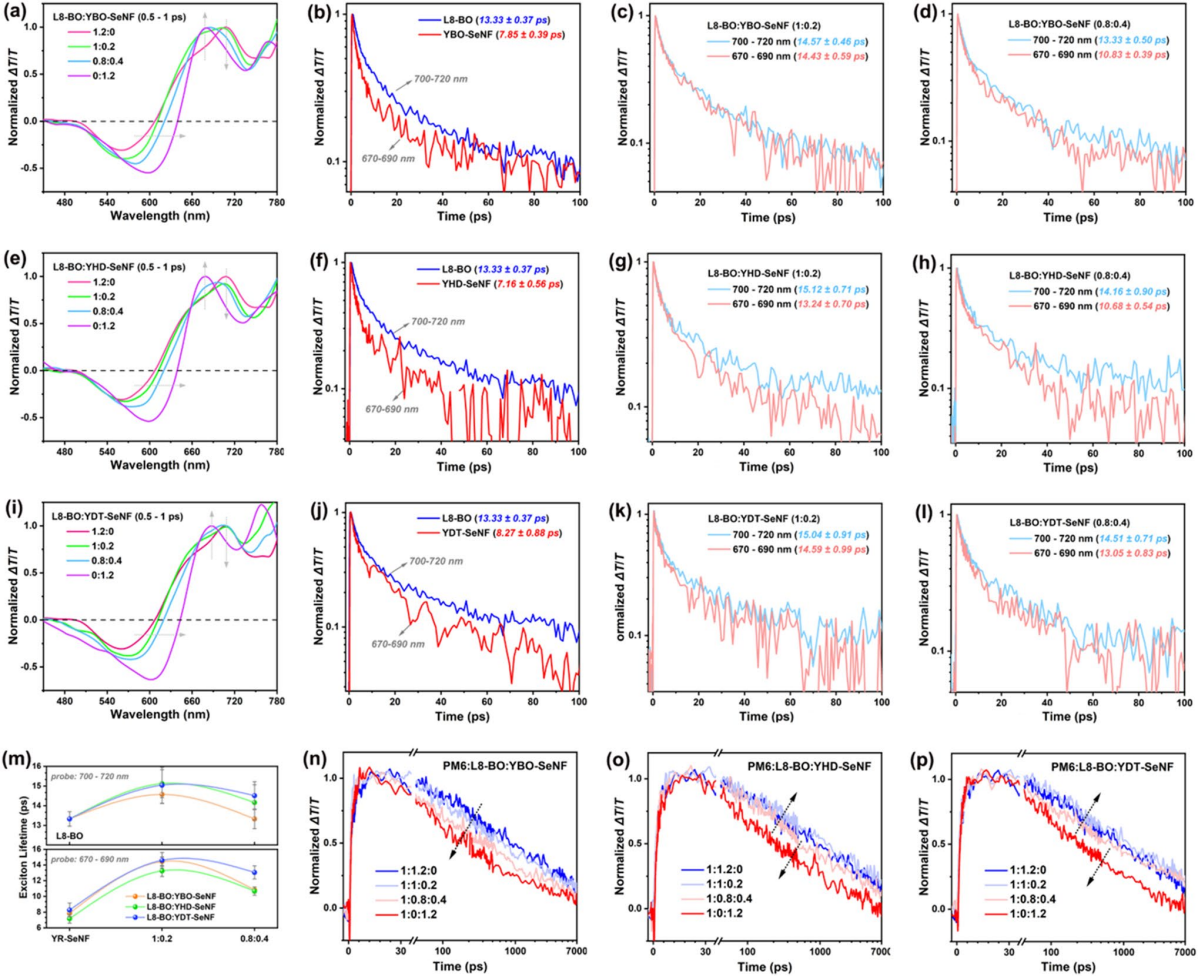
fs-TAS spectra presented in terms of Δ*T/T*. (**a, e**–**i)** Spectral line-cuts of L8-BO:YR-SeNF films with different component ratios at a representative time of 0.5–1 ps. TAS kinetics of singlet excitons in L8-BO:YR-SeNF with different component ratios: **b–d** L8-BO:YBO-SeNF, **f–h** L8-BO:YHD-SeNF, and (j-l) L8-BO:YDT-SeNF, respectively. **m** Summarizes of the identified singlet excitons lifetime monitored of two wavelength ranges of 700–720 nm from L8-BO and 670–690 nm from YR-SeNF series. **n–p** Free charge generation and recombination kinetics in PM6:L8-BO:YR-SeNF with different component ratios

Femtosecond-resolved transient absorption spectra (fs-TAS) of neat films, binary acceptor blends, and ternary blends were measured to study the dynamics of photo-induced charge carriers by using a commonly used excitation fluence of 5 µJ cm^−2^ [58, 59]. According to the absorption spectra of PM6, L8-BO, and YR-SeNF series in neat films, the excitation wavelength selective for the acceptors was determined as 800 nm. The fs-TAS of L8-BO and YR-SeNF series in neat films and the related L8-BO:YR-SeNF (1:0.2 and 0.8:0.4) binary acceptor blends were first measured (Figs. S29-S31). The fs-TAS spectral line-cuts immediate after excitation (i.e., 0.5–1 ps) were chosen in the following discussion to represent the singlet excitons as characterized by ground state bleach (GSB) features similar to the absorption spectra (Fig. S16). As exhibited in Fig. 6a, e, i, the features at 670–690 nm are more dominated by L8-BO singlets while at 700–720 nm are more dominated by the singlets of YR-SeNF series, thereby used as the corresponding probe ranges in the following investigations. As shown in Fig. 6b-d, f–h, j-l, based on either range, the singlet exciton lifetime appears to increase upon blending of acceptors, which could be one of the reasons contributing to the higher *J*_SC_ for ternary OSCs. Figure 6m summarizes the identified singlet excitons lifetime monitored of two wavelength ranges of 700–720 nm (more dominant contribution from L8-BO) and 670–690 nm (more dominant contribution from YR-SeNF series). With introducing appropriate amount of YR-SeNF as a guest acceptor, the resulting exciton lifetime of binary L8-BO:YR-SeNF can be prolonged when compared to either L8-BO or YR-SeNF neat films. Note that YR-SeNF series have red-shifted absorption that is more complementary with L8-BO, so adding more YR-SeNF tends to improve the utilization of solar spectrum. But if exciton lifetimes will be shorter, the advantage might be compensated at certain thresholds. Here, excitons of binary L8-BO:YR-SeNF (0.8:0.4) tend to maintain their lifetime or even slightly increase compared to those at neat films. Consequently, the *J*_SC_ values of ternary OSCs are effectively enhanced.

As depicted in Figs. S32-S34, the fs-TAS of PM6:L8-BO:YR-SeNF ternary blends with different component ratios also were measured. Here, the wavelength range of 560–580 nm representing the dynamics of free charges on the basis of hole polarons was selected. As shown in Fig. 6n-p, the free charge generation kinetics based on the rise of photobleach appears similar for all the blend systems. Adding YBO-SeNF as guest acceptor clearly has negative impacts on sub-ns free charge recombination for all the investigated blend compositions, consequently slightly decreasing FF values can be observed in the related OSCs. Differently, the introduction of YHD-SeNF or YDT-SeNF as the guest component does not harm the sub-ns free charge recombination. In fact, with the appropriate amount of YR-SeNF, ternary blends can even slightly reduce the recombination rate (i.e., longer free charge lifetime), which is beneficial in charge transport, evident from their improved FF in OSCs as well [60]. Combining with the enhanced photon harvesting, an impressively high PCE of approaching 19% was achieved in spin-coating-free and halogen-free solvent processed OSCs.

## Conclusions

A series of newly designed YR-SeNF acceptors with similar NIR-absorption, different crystallinity, packing patterns, and miscibility with polymeric donor are developed. Introducing YR-SeNF guest acceptors associated with tailored *N*-alkyl chains into PM6:L8-BO host system well manipulate active layer morphologies, such as molecular packing, crystallinity, and vertical distribution, as well as improving charge transfer dynamics and stability in OSCs. As a result, the OSCs with excellent stability achieve a PCE approaching 19%, which is the highest value among OSCs with active layers processed from halogen-free solvents and/or spin-coating-free technologies. As such, we believe that our strategy provides a feasible method to print eco-friendly, high-efficiency, and operating stable OSCs, which paves the way for industrial development.

### Supplementary Information

Below is the link to the electronic supplementary material.

Supplementary file1 (PDF 5823 KB)
