# Supplementary material for: Green-Solvent Processed Blade-Coating Organic Solar Cells with an Efficiency Approaching 19% Enabled by Alkyl-Tailored Acceptors
Source: Nanomicro Lett. 2023 Nov 2;15:241. doi: 10.1007/s40820-023-01208-0 (PMC10622389; doi:10.1007/s40820-023-01208-0)
Supplement: Supplementary file 1 — Supplementary file1 (PDF 5823 KB) [file 40820_2023_1208_MOESM1_ESM.pdf]

Supporting Information for

# Green-Solvent Processed Blade-Coating Organic Solar Cells with an Efficiency Approaching 19% Enabled by Alkyl-Tailored Acceptors

Hairui Bai[1], Ruijie Ma[4, *], Wenyan Su[5, 9, *], Top Archie Dela Peña[6, 7], Tengfei Li[1], Lingxiao Tang[1], Jie Yang[8] Bin Hu[10], Yilin Wang[1], Zhaozhao Bi,[1], Yueling Su[9], Qi Wei[6], Qiang Wu[1, *], Yuwei Duan[9], Yuxiang Li[5], Jiaying Wu[7], Zicheng Ding[9], Xunfan Liao[3], Yinjuan Huang[1], Chao Gao[11], Guanghao Lu[10], Mingjie Li[6], Weiguo Zhu[2], Gang Li [4, *], Qunping Fan[1, 2, 3, *], and Wei Ma[1, *]

[1] State Key Laboratory for Mechanical Behavior of Materials, Xi’an Jiaotong University, Xi’an 710049, P. R. China

[2] Jiangsu Engineering Research Center of Light-Electricity-Heat Energy-Converting Materials and Applications, School of Materials Science and Engineering, and Changzhou University, Changzhou 213164, P. R. China

[3] Key Lab of Fluorine and Silicon for Energy Materials and Chemistry of Ministry of Education/National Engineering Research Center for Carbohydrate Synthesis, Jiangxi Normal University, 99 Ziyang Avenue, Nanchang 330022, P. R. China

[4] Department of Electronic and Information Engineering, Research Institute for Smart Energy (RISE), The Hong Kong Polytechnic University, Kowloon 999077, Hong Kong, P. R. China

[5] School of Materials Science and Engineering, Xi’an University of Science and Technology, Xi’an 710054, P. R. China

[6] Department of Applied Physics, The Hong Kong Polytechnic University, Kowloon, Hong Kong 999077, P. R. China

[7] Advanced Materials Thrust, Function Hub, The Hong Kong University of Science and Technology, Nansha, Guangzhou, P. R. China

[8] School of Chemistry and Chemical Engineering, Beijing Institute of Technology, Beijing 100081, P. R. China

[9] Key Laboratory of Applied Surface and Colloid Chemistry, Ministry of Education, Shaanxi Key Laboratory for Advanced Energy Devices, Shaanxi Engineering Lab for Advanced Energy Technology School of Materials Science and Engineering, Shaanxi Normal University, Xi’an 710119, P. R. China

[10] Frontier Institute of Science and Technology, Xi’an Jiaotong University, Xi’an 710054, P. R. China

[11] Xi'an Key Laboratory of Liquid Crystal and Organic Photovoltaic Materials, State Key Laboratory of Fluorine & Nitrogen Chemicals, Xi'an Modern Chemistry Research Institute, Xi'an 710065, P. R. China

*Corresponding authors. E-mail: ruijie.ma@polyu.edu.hk (Ruijie Ma); suwy1027@xust.edu.cn (Wenyan Su); wuqiang@xjtu.edu.cn (Qiang Wu); gang.w.li@polyu.edu.hk (Gang Li); qunping@xjtu.edu.cn (Qunping Fan); msewma@xjtu.edu.cn (Wei Ma)

## S1 Experimental Section

***Materials*****:** Monomers YBO-CHO [S1], BPSe [S2], and YDT-CHO[S3], Y-series small-molecule acceptor (Y-SMA) L8-BO[S4], polymer donor PM6 with a *M*n of 31.7 kDa (Lot# YMA08A in Solarmer Materials Inc) [S5], and end-group NpIC-2F [S6] were synthesized according to the previous reports. YR-SeNF series (YBO-SeNF, YHD-SeNF, and YDT-SeNF) were developed according to the following procedures:

***YBO-SeNF***: In a dry 100 mL flask, compounds of YBO-2CHO (0.10 g, 0.081 mmol), NpIC-2F (0.080 g, 0.284 mmol), and pyridine (0.1 mL) were added to 10 mL of degassed chloroform under argon and stirred vigorously at 65 °C for 12 h. Then the mixture was poured into methanol (100 mL) followed by precipitation, and the sediments were collected. The resulting crude compound was purified by column chromatography on silica gel with hexane/$CH_2Cl_2$ (*v*/*v*=2:1) as eluent and then it was furtherly purified by crystallized two times using a mix solvent of chloroform/acetone (*v*/*v*=1:1) to give YBO-SeNF as a black solid (0.097 g, 68%). $^1$H NMR (600 MHz, $CDCl_3$, TMS), (ppm): $\delta$ 9.31 (s, 2H), 9.02 (s, 2H), 8.31 (s, 2H), 7.83-7.77 (m, 4H), 4.76 (t, $J$ = 3.0 Hz, 4H), 3.23 (m, 4H), 2.25-2.22 (m, 2H), 1.86 (m, 4H), 1.55-1.47 (m, 4H), 1.35 (m, 4H), 1.26 (m, 24H), 1.20-1.09 (m, 8H), 1.09-0.91 (m, 12H), 0.85 (m, 12H), 0.76-0.65 (m, 18H). $^{13}$C NMR (600 MHz, $CDCl_3$, TMS), (ppm): $\delta$ 188.54, 159.94, 157.01, 147.78, 147.47, 142.17, 138.37, 138.18, 135.25, 134.92, 134.26, 133.47, 133.42, 132.47, 132.42, 130.09, 125.48, 122.30, 121.28, 116.56, 116.45, 116.09, 115.98, 115.70, 115.43, 113.71, 66.92, 55.82, 39.03, 31.92, 31.63, 31.58, 31.36, 30.47, 30.36, 29.97, 29.68, 29.64, 29.54, 29.47, 29.41, 29.35, 28.04, 27.92, 25.52, 25.39, 22.89, 22.83, 22.69, 22.53, 22.51, 14.13, 14.07, 14.03, 13.82, 13.79. MALDI-TOF-MS m/z: $[M+Na]^+$ calcd. for $C_{98}H_{106}F_4N_8O_2S_3Se_2Na^+$, 1758.58674+$Na^+$, found 1781.58603.

***BPSe-HD***: In a dry 100 mL flask, compounds of BPSe (0.85 g, 1.01 mmol), $K_2CO_3$ (1.40 g, 10.1 mmol), KI (3.35 g, 20.2 mmol), and 1-bromo-2-hexyl-decane (1.85 g, 6.06 mmol) were added to 10 mL of degassed DMF under argon and stirred vigorously at 100 °C for 12 h. The reaction was quenched by water and extracted with $CH_2Cl_2$. The combined extracts were washed with brine, dried over anhydrous $MgSO_4$, and then filtered and collected solvent. The solvent was removed by rotary evaporation to give the crude product as an orange oil, which was further purified by column chromatography on silica gel with hexane/$CH_2Cl_2$ (v/v, 8:1) as eluent to afford BPSe-HD as an orange viscous liquid (0.80 g, 61.4%). $^1$H NMR (600 MHz, $CDCl_3$, TMS), (ppm): $\delta$ 7.53 (s, 2H), 4.55 (d, $J$ = 7.6 Hz, 4H), 2.85-2.72 (m, 4H), 2.16-1.98 (m, 2H), 1.95-1.75 (m, 4H), 1.45-1.27 (m, 34H), 1.20-0.79 (m, 44H), 0.69-0.65 (m, 18H).

***YHD-CHO***: In an ice bath, phosphorus oxychloride ($POCl_3$, 3 mL) was dropwise added into a solution of anhydrous DMF (3 mL) and kept 10 minutes under the protection of argon, and then kept additional 2 hours at room temperature under stirring. After that, BPSe-HD (0.40 g, 0.31 mmol) dissolved in chloroform (10 mL) was added into the reaction mixture and then heated at 65 ℃ for overnight, cooled to room temperature, quenched with saturated aqueous solution of sodium acetate, and extracted with $CH_2Cl_2$. The combined extracts were washed with brine, dried over anhydrous $MgSO_4$, and then filtered and collected solvent. The solvent was removed by rotary evaporation to yield the crude product, which was then purified by column chromatography on silica gel with hexane/$CH_2Cl_2$ (v/v, 2:1) as eluent to afford YHD-CHO as a red viscous liquid (0.36 g, 86%). $^1$H NMR (600 MHz, $CDCl_3$, TMS), (ppm): $\delta$ 10.04 (s, 2H), 4.58 (d, $J$ = 7.6 Hz, 4H), 3.18 (t, $J$ = 7.6 Hz, 4H), 2.09-1.98 (m, 2H), 1.98-1.83 (m, 4H), 1.51-1.26 (m, 34H), 1.19-0.79 (m, 44H), 0.69-0.65 (m, 18H).

***YHD-SeNF***: In a dry 100 mL flask, compounds of YHD-2CHO (0.090 g, 0.067 mmol), NpIC-2F (0.067 g, 0.234 mmol), and pyridine (0.1 mL) were added to 10 mL of degassed chloroform under argon and stirred vigorously at 65 °C for 12 h. Then the mixture was poured

into methanol (100 mL) followed by precipitation, and the sediments were collected. The resulting crude compound was purified by column chromatography on silica gel with hexane/$CH_2Cl_2$ (*v*/*v*=2:1) as eluent and then it was furtherly purified by crystallized two times using a mix solvent of chloroform/acetone (*v*/*v*=1:1) to give YHD-SeNF as a black solid (0.089 g, 71%). $^1$H NMR (600 MHz, $CDCl_3$, TMS), (ppm): $\delta$ 9.30 (s, 2H), 9.01 (s, 2H), 8.31 (s, 2H), 7.83-7.75 (m, 4H), 4.77 (d, *J* = 7.3 Hz, 4H), 3.22 (t, *J* = 7.5 Hz, 4H), 2.35-2.15 (m, 2H), 1.97-1.73 (m, 4H), 1.54-1.25 (m, 34H), 1.19-1.03 (m, 40), 0.88-0.71 (m, 22H). $^{13}$C NMR (600 MHz, $CDCl_3$, TMS), (ppm): $\delta$ 188.48, 159.63, 156.94, 147.88, 147.38, 142.27, 138.60, 137.99, 135.19, 134.98, 134.25, 133.47, 133.39, 132.53, 132.44, 130.02, 125.38, 123.30, 121.10, 116.53, 116.37, 116.22, 116.05, 115.75, 115.45, 113.84, 66.86, 55.99, 39.24, 32.00, 31.96, 31.73, 31.44, 30.77, 30.10, 30.00, 29.79, 29.73, 29.67, 29.51, 29.45, 29.35, 25.89, 25.82, 22.77, 22.75, 22.66, 14.24, 14.21. MALDI-TOF-MS m/z: $[M]^+$ calcd. for $C_{106}H_{122}F_4N_8O_2S_3Se_2$, 1871.71530, found 1871.72891.

***YDT-SeNF***: In a dry 100 mL flask, compounds of YDT-2CHO (0.080 g, 0.051 mmol), NpIC-2F (0.050 g, 0.178 mmol), and pyridine (0.1 mL) were added to 10 mL of degassed chloroform under argon and stirred vigorously at 65 °C for 12 h. Then the mixture was poured into methanol (100 mL) followed by precipitation, and the sediments were collected. The resulting crude compound was purified by column chromatography on silica gel with hexane/$CH_2Cl_2$ (*v*/*v*=2:1) as eluent and then it was furtherly purified by crystallized two times using a mix solvent of chloroform/acetone (*v*/*v*=1:1) to give YDT-SeNF as a black solid (0.078 g, 73%). $^1$H NMR (600 MHz, $CDCl_3$, TMS), (ppm): $\delta$ 9.31 (s, 2H), 9.03 (s, 2H), 8.31 (s, 2H), 7.83-7.75 (m, 4H), 4.76 (d, *J* = 7.0 Hz, 4H), 3.22 (t, *J* = 7.4 Hz, 4H), 2.39-2.16 (m, 2H), 1.98-1.76 (m, 4H), 1.55-1.02 (m, 98H), 0.88-0.79 (m, 22H). $^{13}$C NMR (600 MHz, $CDCl_3$, TMS), (ppm): $\delta$ 188.54, 159.85, 157.03, 147.86, 147.51, 142.25, 138.54, 138.13, 135.28, 135.02, 134.36, 133.54, 133.46, 132.53, 132.46, 130.17, 125.51, 123.31, 121.24, 116.66, 116.46, 116.17, 115.99, 115.74, 115.48, 113.82, 66.92, 55.86, 39.08, 32.05, 32.03, 31.75, 31.45, 30.62, 30.12, 29.92, 29.84, 29.75, 29.69, 29.57, 29.51, 29.47, 25.76, 25.73, 22.79, 14.21. MALDI-TOF-MS m/z: $[M+Na]^+$ calcd. for $C_{122}H_{154}F_4N_8O_2S_3Se_2Na^+$, 2095.96570+$Na^+$, found 2117.95845.

***Instruments and Measurements***: $^1$H NMR and $^{13}$C NMR spectra were collected with a Bruker 600 MHz ASCEND ADVANCE III HD spectrometer in $CDCl_3$. The UV-vis absorption spectra were measured by a Shimadzu UV-3600 Plus Spectrophotometer. Cyclic voltammetry was probed on a CHI660D electrochemical workstation in an anhydrous acetonitrile solution of tetra-n-butylammonium hexafluoro-phosphate ($Bu_4NPF_6$) (0.1 M) with a scan rate of 50 mV $s^{-1}$. A conventional three-electrode cell was used with a platinum plate working electrode, a platinum wire counter-electrode, and an Ag/AgCl reference electrode. Ferrocene/Ferrocenium ($Fc/Fc^+$) used as the internal standard and their energy levels are assumed at -4.8 eV relative to vacuum. The corresponding HOMO and LUMO levels are estimated according to the empirical equations: $E_{HOMO}= -(E_{ox}+4.28)$ eV and $E_{LUMO}= -(E_{red}+4.28)$ eV. Atomic force microscopy (AFM) characterization was scanned by Veeco INNOVA Atomic Force Microscope using a tapping mode. Transmission electron microscopy (TEM) was performed using a Tecnai G2 F20 S-TWIN instrument at 200 kV accelerating voltage. TEM was performed using a Tecnai G2 F20 S-TWIN instrument at 200 kV accelerating voltage, in which the blend films were prepared using a processing technique, as following: first, the blend films were blade-coating on the PEDOT:PSS/ITO substrates; second, the resulting blend film/PEDOT:PSS/ITO substrates were submerged in deionized water to make these blend films float onto the air-water interface; finally, the floated blend films were taken up on unsupported 200 mesh copper grids for a TEM measurement. GIWAXS measurements were performed at beamline 7.3.3 at the Advanced Light Source.

Samples were prepared on Si substrates using identical blend solutions as those used in devices. The 10 keV X-ray beam was incident at a grazing angle of 0.11-0.15°, selected to maximize the scattering intensity from the samples. The scattered x-rays were detected using a Dectris Pilatus 2M photon counting detector. The crystal coherence length (CCL) was defined as CCL = 0.9×(2π/FWHM) (Å), where FWHM is the full width at half maximum of the corresponding diffraction peak.

***Computational Details***: To gain a deeper understanding of the effect of alkyl-chain engineering on intermolecular interactions and packing patterns, the crystal structures of the studied acceptors were predicted by carrying out molecular dynamics (MD) simulations. Firstly, the single molecule was optimized using the Dmol3 program with a fine accuracy setting. Additionally, electrostatic potential and population analysis were performed. Subsequently, crystal structure prediction was carried out using the Perdew-Burke-Ernzerhof (PBE) exchange-correlation energy functional and the Dreiding force field. This process was conducted in the polymorph module, which has proven to be a valuable method for predicting crystal structures.[S7] All calculations were performed using Materials Studio (One Molecular Simulation Software, https://www.accelrys.com).

***Mobility Measurement***: The electron and hole mobilities of blends were measured by using a space-charge limited current (SCLC) method. The electron-only device with a structure of ITO/ZnO/active layer/PFN-Br-MA/Ag and the hole-only device with an architecture of ITO/2PACz/active layer/$MoO_x$/Al were fabricated. The mobility was determined by fitting the dark current to the model of a single carrier SCLC, described by the equation: $J=9\varepsilon_0\varepsilon_r\mu V^2/8L^3$, where $J$ is the current density, $\varepsilon_0$ is the permittivity of free space, $\varepsilon_r$ is the relative dielectric constant of the transport medium, $\mu$ is the charge mobility and L is the thickness of the active layer. $V=V_{app} - V_{bi}$, where $V_{app}$ is the applied voltage, $V_{bi}$ is the offset voltage ($V_{bi}$ is 0 V here). The electron mobility can be calculated from the slope of the $J_{0.5}$~$V$ curves.

***Transient Absorption Spectroscopy (TAS)***: TAS was measured with an amplified Ti:sapphire

femtosecond laser (800 nm wavelength, 50 fs, 1 kHz repetition; Coherent Libra) and a Helios pump/probe setup (Ultrafast Systems). The 400 nm pump pulses with a pump fluence of 0.5 or < 0.3 μJ/cm$^2$ were obtained by frequency doubling the 800 nm fundamental regenerative amplifier output. The white-light continuum probe pulses were generated by focusing a small portion of the regenerative amplifier's fundamental 800 nm laser pulses into a 2 mm sapphire crystal.

## S2 Supplementary Figures and Tables

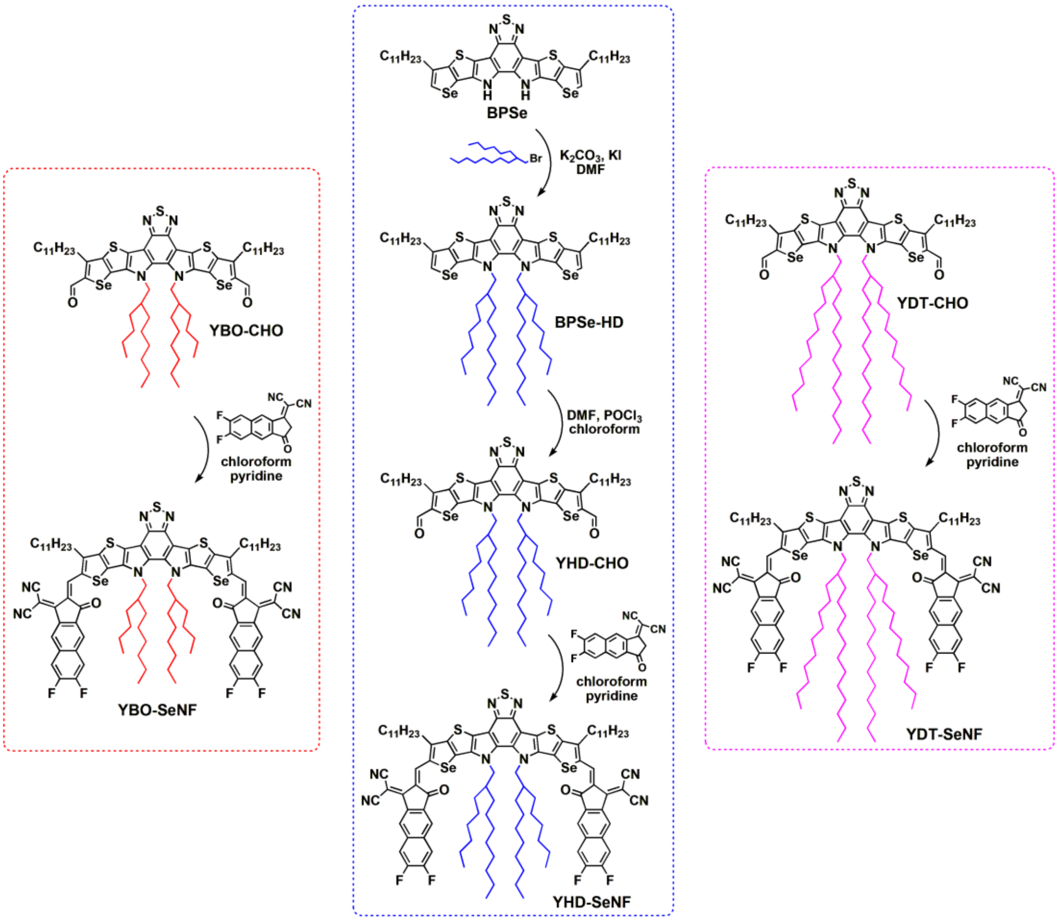


**Fig. S1** The synthetic routes of YBO-SeNF, YHD-SeNF, and YDT-SeNF

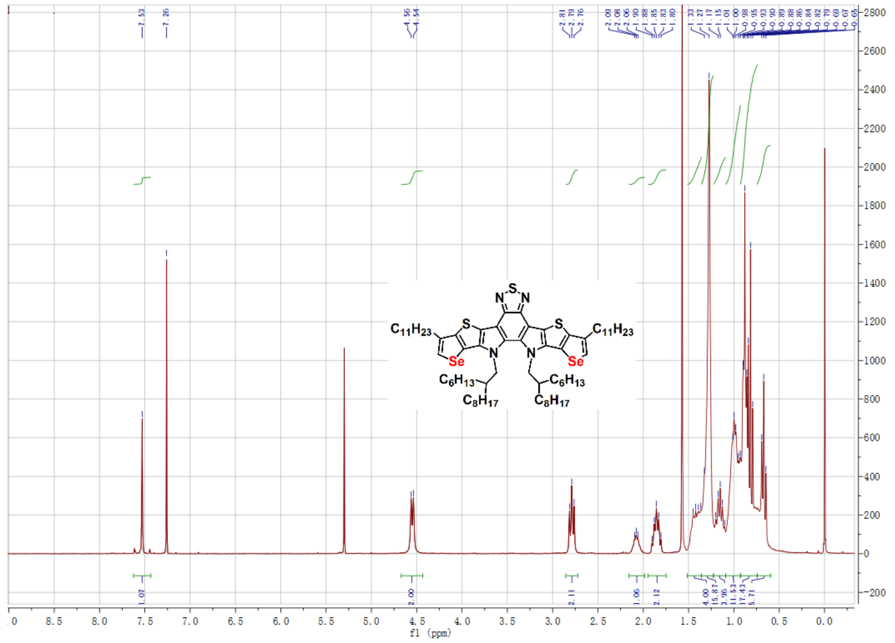


**Fig. S2** $^{1}$H NMR image of BPSe-HD

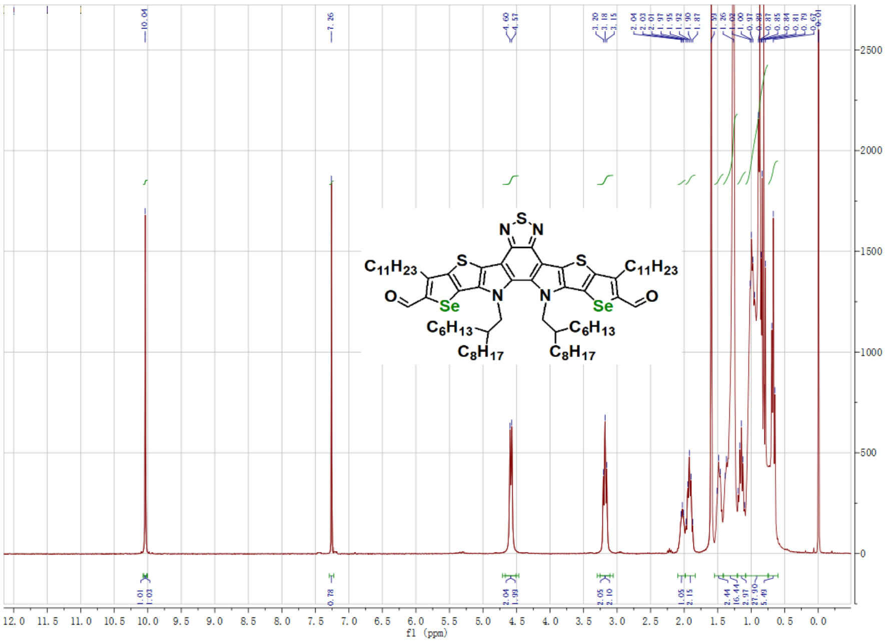


**Fig. S3** $^{1}$H NMR image of YHD-CHO

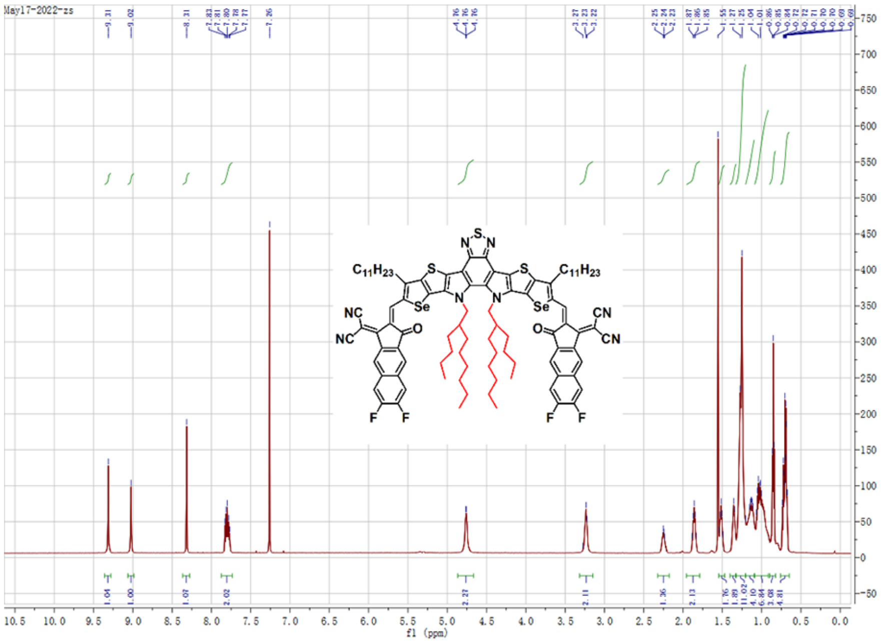


**Fig. S4** $^{1}$H NMR image of YBO-SeNF

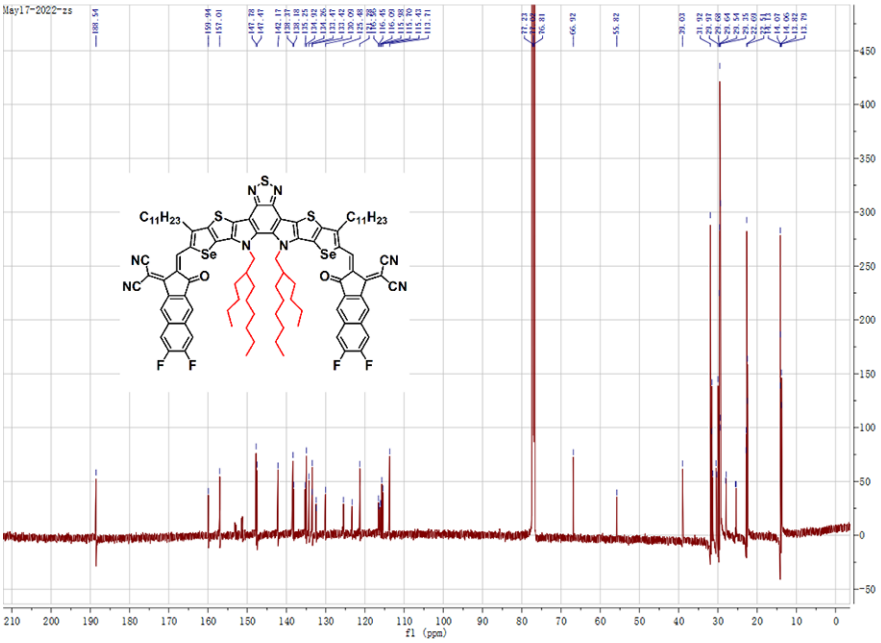


**Fig. S5** $^{13}C$ NMR image of YBO-SeNF

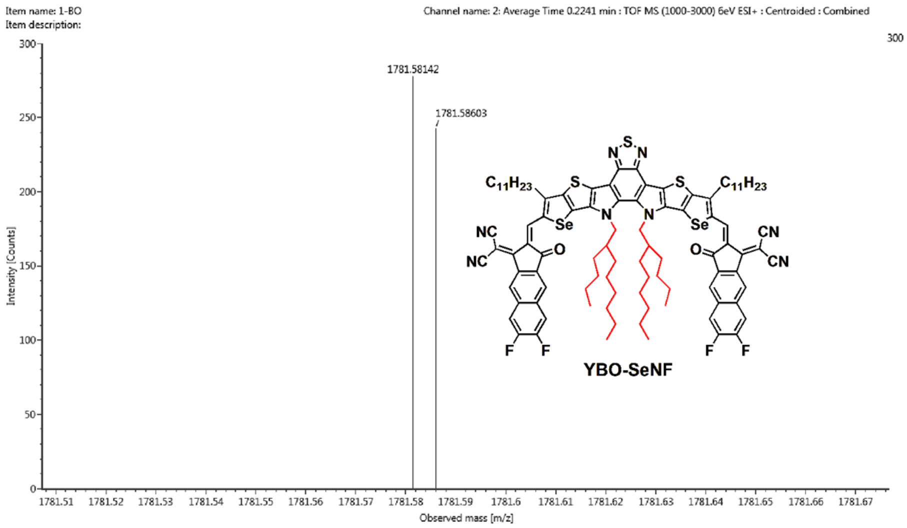


**Fig. S6** MS image of YBO-SeNF

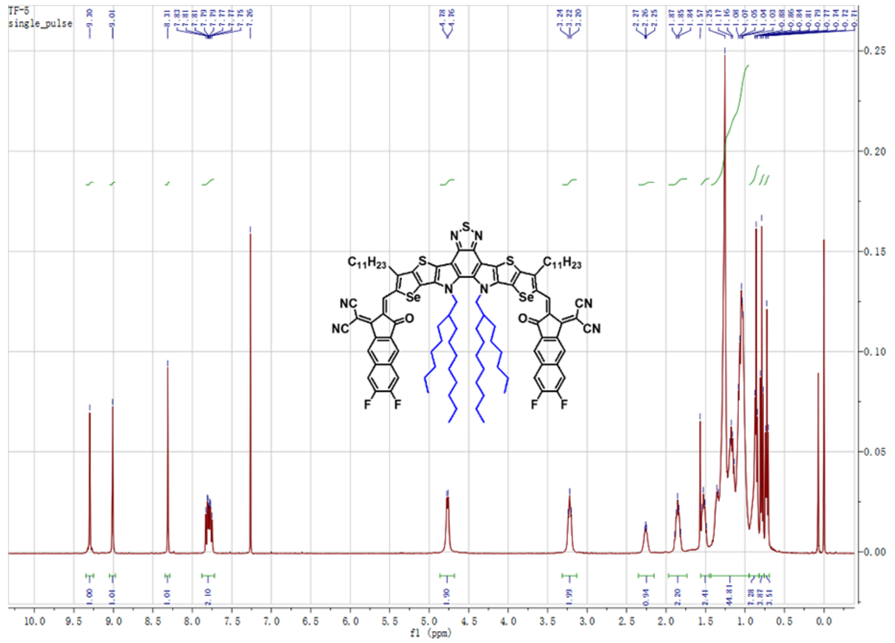

**Fig. S7** $^{1}H$ NMR image of YHD-SeNF

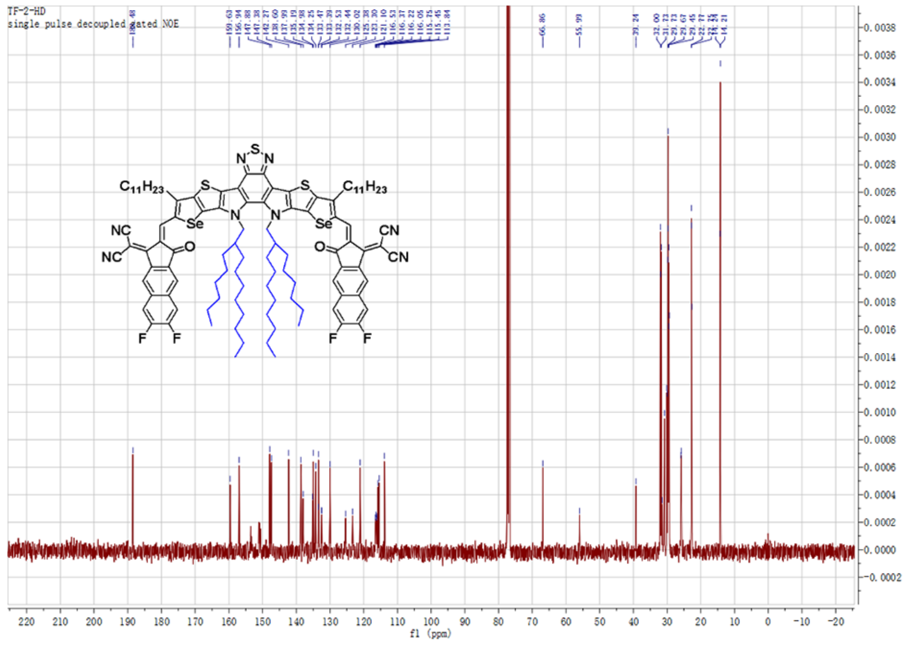

**Fig. S8** $^{13}C$ NMR image of YHD-SeNF

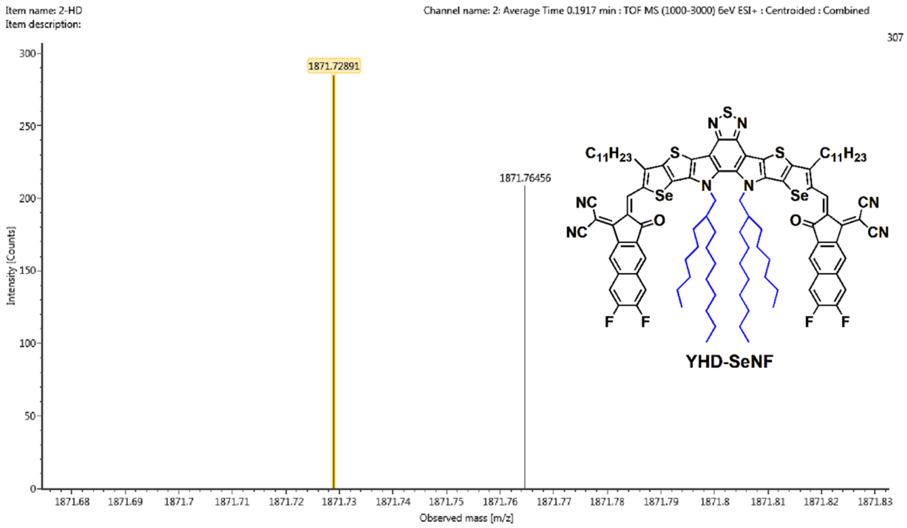


**Fig. S9** MS image of YHD-SeNF

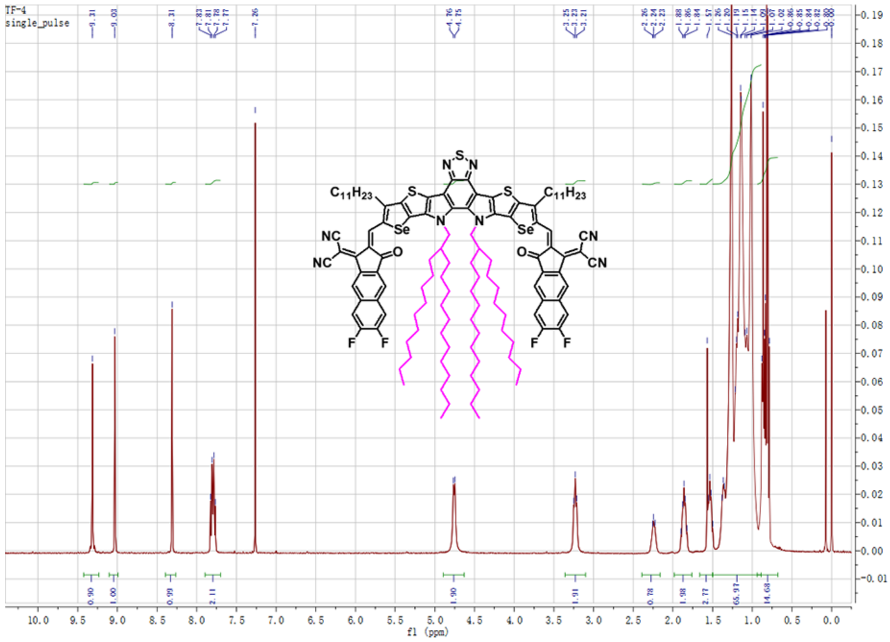


**Fig. S10** $^{1}$H NMR image of YDT-SeNF

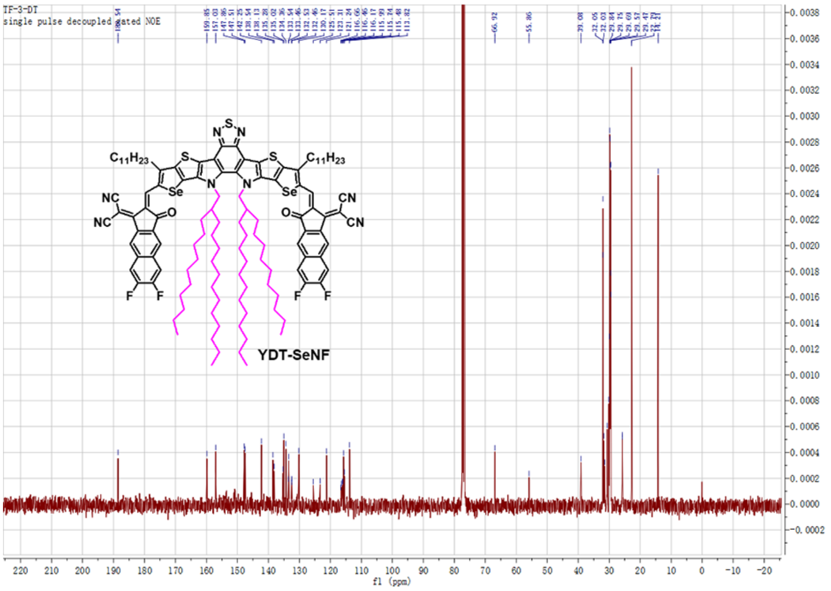


**Fig. S11** $^{13}$C NMR image of YDT-SeNF

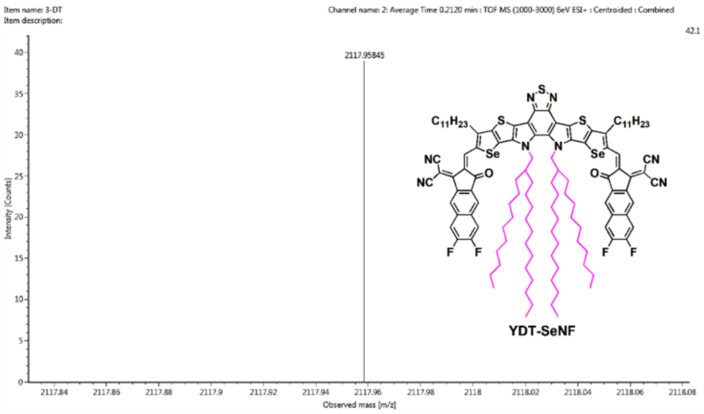


**Fig. S12** MS image of YDT-SeNF

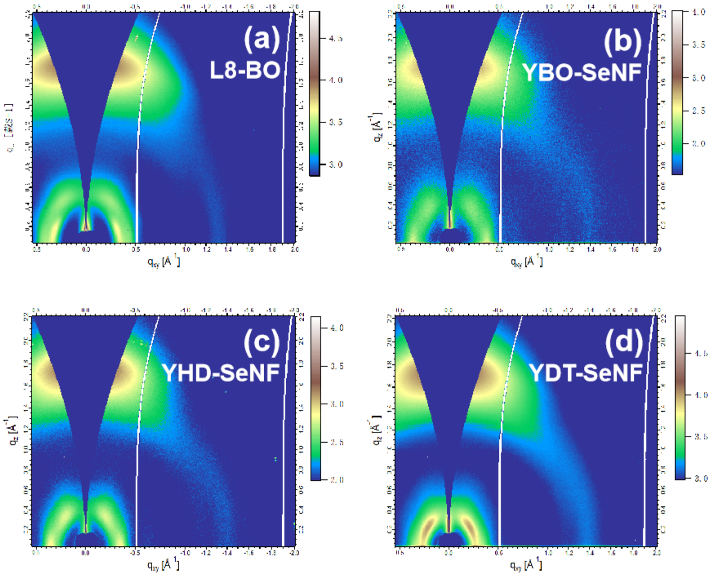


**Fig. S13** 2D-GIWAXS images of blade-coating neat films: (**a**) L8-BO, (**b**) YBO-SeNF, (**c**) YHD-SeNF, (**d**) YDT-SeNF, respectively

**Table S1** GIWAXS test performance parameters of L8-BO and YR-SeNF neat films

| | in plane | | | | | |
|---|---|---|---|---|---|---|
| | location (Å$^{-1}$) | d-spacing (Å) | CCL (Å) | location (Å$^{-1}$) | d-spacing (Å) | CCL (Å) |
| L8-BO | 0.346 | 18.184 | 76.960 | 0.431 | 14.590 | 53.601 |
| YBO-SeNF | 0.376 | 16.696 | 53.990 | | | |
| YHD-SeNF | 0.352 | 17.853 | 53.464 | | | |
| YDT-SeNF | 0.267 | 23.565 | 75.185 | 0.342 | 18.355 | 59.385 |
| | out of plane | | | | | |
| | location (Å$^{-1}$) | d-spacing (Å) | CCL (Å) | location (Å$^{-1}$) | d-spacing (Å) | CCL (Å) |
| L8-BO | 1.734 | 3.624 | 16.472 | | | |
| YBO-SeNF | 1.717 | 3.659 | 16.572 | | | |
| YHD-SeNF | 1.701 | 3.694 | 16.269 | | | |
| YDT-SeNF | 1.686 | 3.726 | 15.010 | | | |

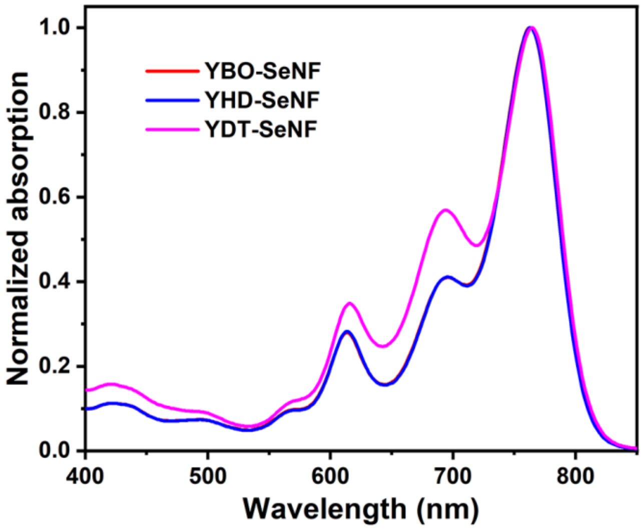


**Fig. S14** Normalized absorption spectra of YR-SeNF series in dilute *o*-xylene solutions

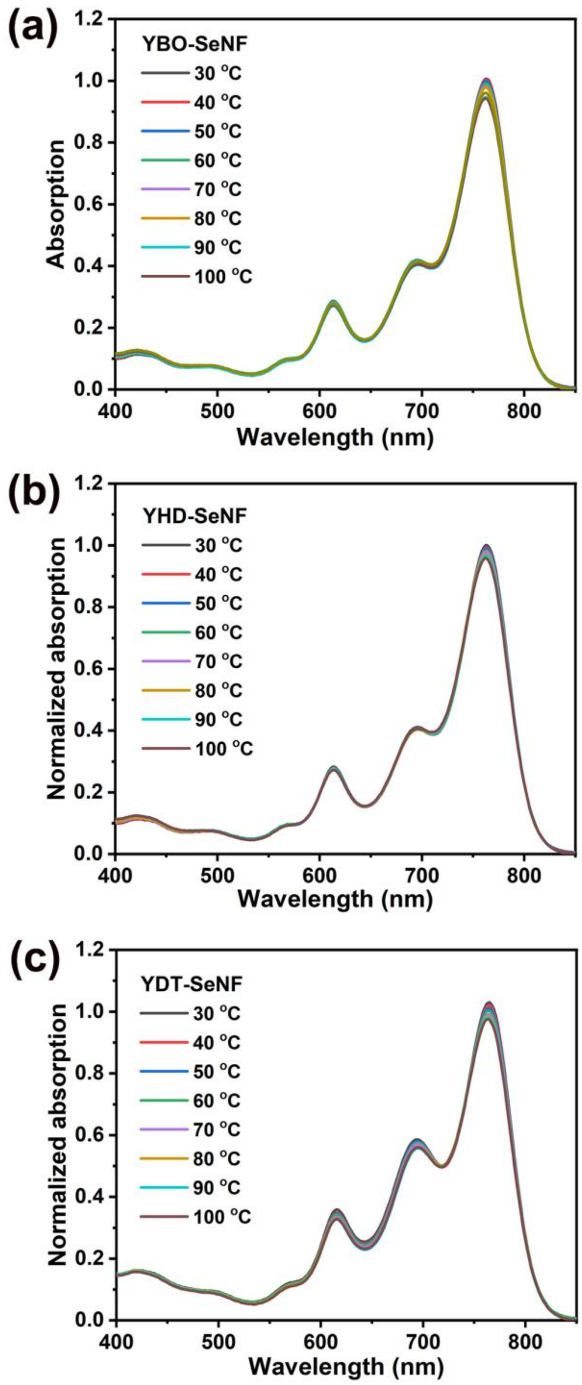


**Fig. S15.** Absorption spectra of YR-SeNF series in dilute *o*-xylene solutions with different temperatures: (**a**) YBO-SeNF, (**b**) YHD-SeNF, and (**c**) YDT-SeNF, respectively

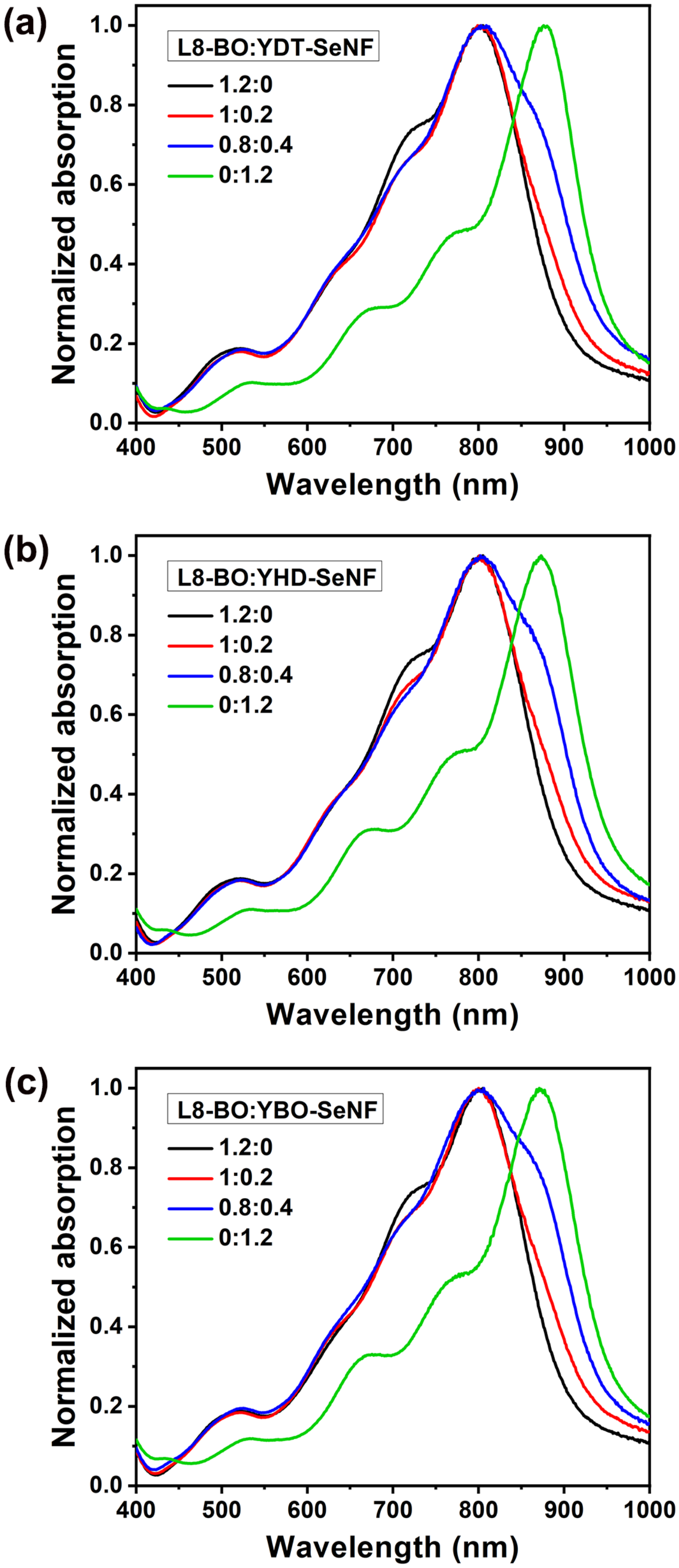


**Fig. S16** Normalized absorption spectra of binary acceptor blends with different ratios: (**a**) L8-BO:YBO-SeNF, (**b**) L8-BO:YHD-SeNF, and (**c**) L8-BO:YDT-SeNF

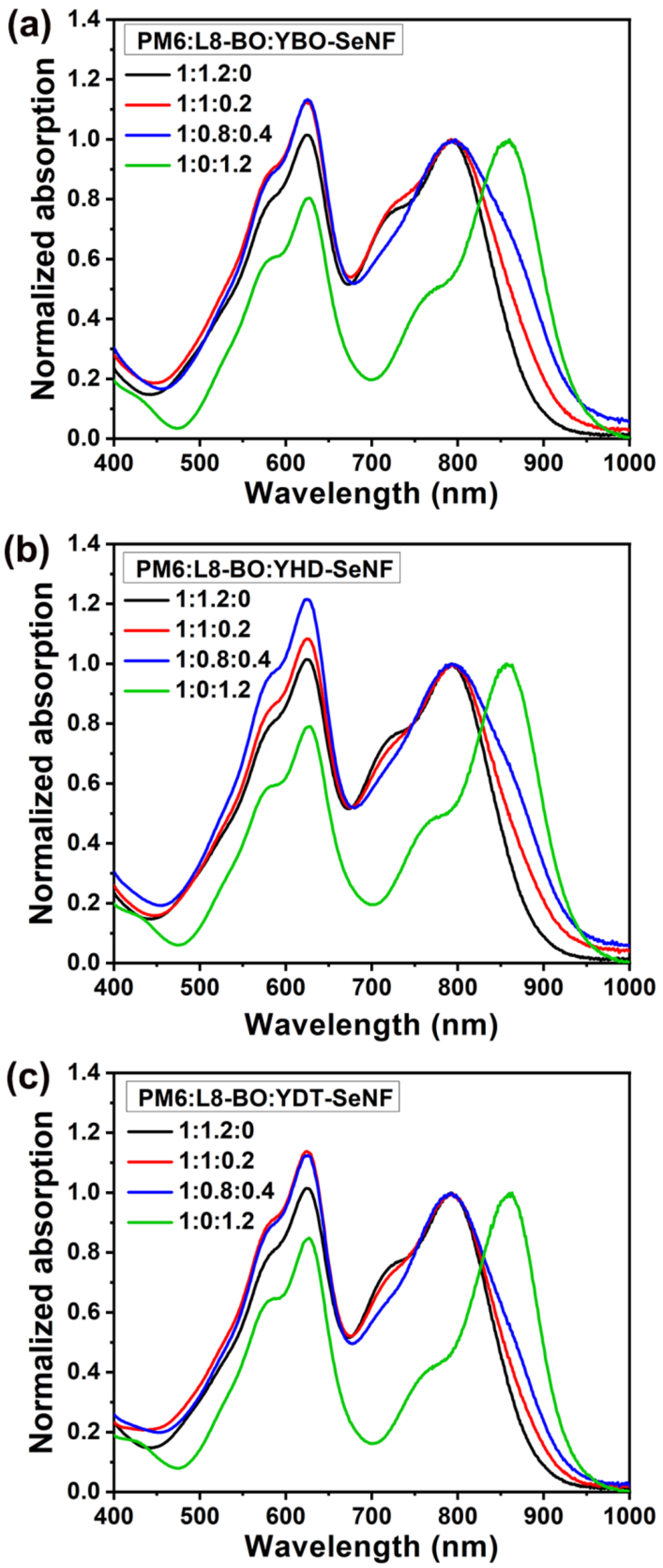


**Fig. S17.** Normalized absorption spectra of ternary active layers with different component ratios: (**a**) PM6:L8-BO:YBO-SeNF, (**b**) PM6:L8-BO:YHD-SeNF, and (**c**) PM6:L8-BO:YDT-SeNF

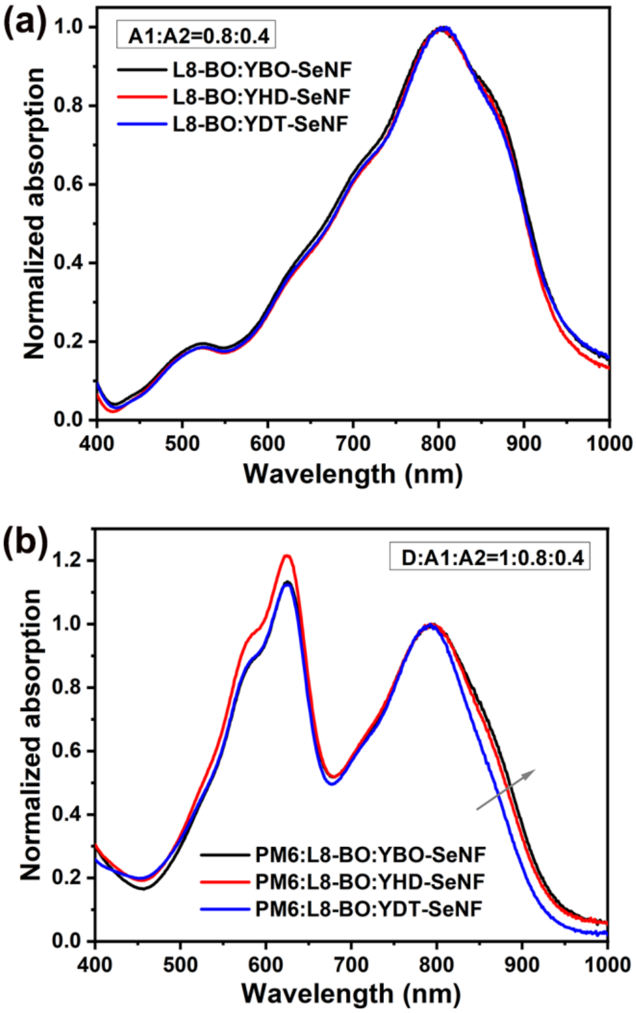


**Fig. S18** Normalized absorption spectra of blend films: (**a**) binary L8-BO:YR-SeNF with a ratio of 0.8:0.4 and (**b**) ternary PM6:L8-BO:YR-SeNF with a ratio of 1:0.8:0.4

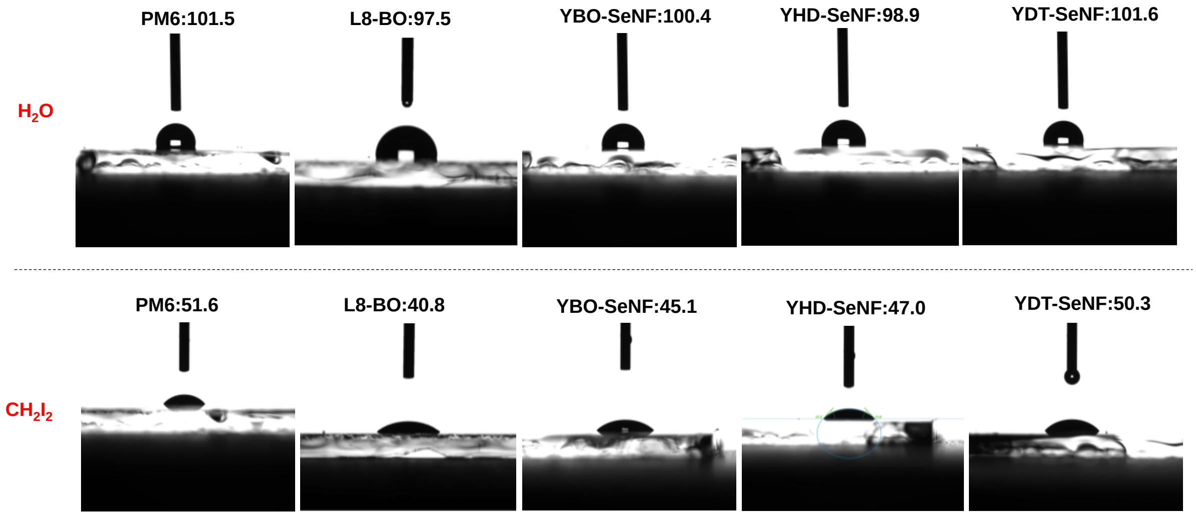


**Fig. S19** Contact angle images of active layer materials in neat films on water ($H_2O$) and diiodomethane ($CH_2I_2$)

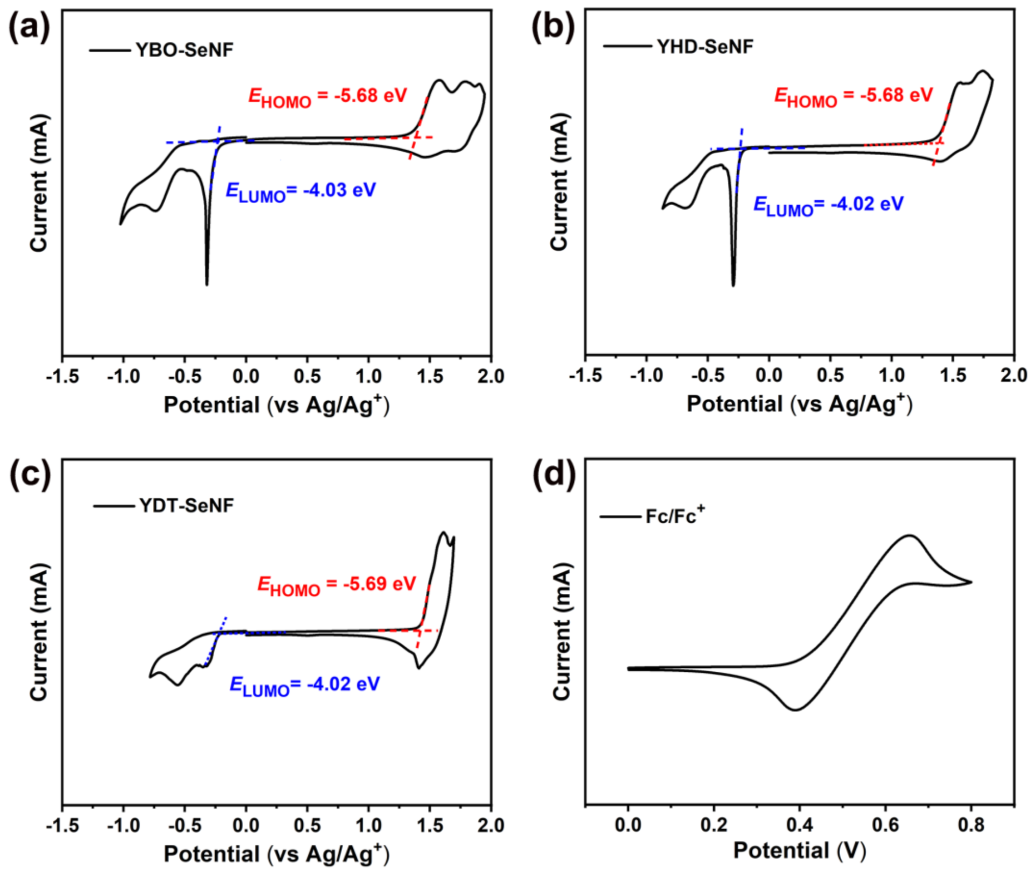


**Fig. S20** Cyclic voltammograms of YR-SeNF neat films on a glassy carbon electrode measured in a 0.1 mol/L $Bu_4NPF_6$ acetonitrile solution at a scan rate of 50 mV/s

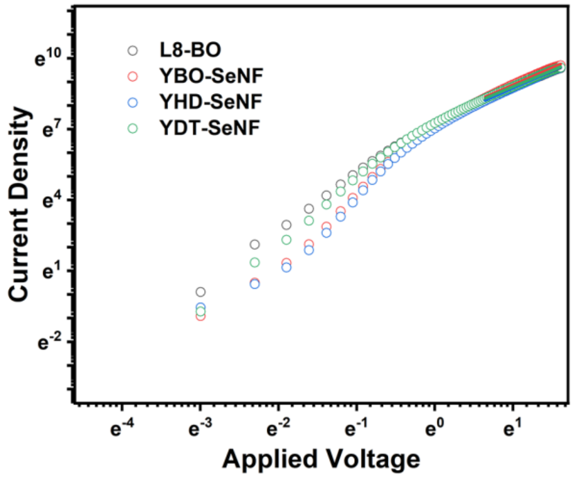


**Fig. S21** Current density-applied voltage characteristics for the electron-only devices based on L8-BO and YR-SeNF neat films

**Table S2** The charge mobilities of the electron-only devices according to the SCLC method

| | $\mu_e$ (cm$^2$ V$^{-1}$ s$^{-1}$) |
|---|---|
| L8-BO | $1.82 \times 10^{-3}$ |
| YBO-SeNF | $1.94 \times 10^{-3}$ |
| YHD-SeNF | $1.65 \times 10^{-3}$ |
| YDT-SeNF | $1.90 \times 10^{-3}$ |

**Table S3** Photovoltaic parameters of the OSCs based on active layers processed from halogen-free solvents

| Active layers | Solvent | $V_{OC}$ [V] | $J_{SC}$ [mA cm$^{-2}$] | FF | PCE [%] | Refs. |
|---|---|---|---|---|---|---|
| PM6:L8-BO:YBO-SeNF (1:1:0.2) | *o*-xylene | 0.869 | 27.3 | 0.776 | 18.4 | **This Work** |
| PM6:L8-BO:YBO-SeNF (1:0.8:0.4) | *o*-xylene | 0.861 | 27.5 | 0.772 | 18.2 | **This Work** |
| PM6:L8-BO:YHD-SeNF (1:1:0.2) | *o*-xylene | 0.874 | 27.3 | 0.789 | 18.8 | **This Work** |
| PM6:L8-BO:YHD-SeNF (1:0.8:0.4) | *o*-xylene | 0.866 | 28.0 | 0.775 | 18.8 | **This Work** |
| PM6:L8-BO:YDT-SeNF (1:1:0.2) | *o*-xylene | 0.871 | 26.9 | 0.788 | 18.5 | **This Work** |
| PM6:L8-BO:YDT-SeNF (1:0.8:0.4) | *o*-xylene | 0.864 | 27.8 | 0.775 | 18.6 | **This Work** |
| PffBT4T-2DT:EH-IDTBR | Mesitylene | 1.02 | 17.2 | 63.0 | 11.1 | S8 |
| PBQ-4F:ITIC | THF | 0.95 | 17.87 | 66.8 | 11.34 | S9 |
| FTAZ:IT-M | Toluene | 0.95 | 16.8 | 66.1 | 10.6 | S10 |
| PBDTS-TDZ:ITIC | *o*-xylene | 1.1 | 17.78 | 65.4 | 12.8 | S11 |
| PBTA-TF:IT-M | *o*-xylene | 0.97 | 17.76 | 71.0 | 12.2 | S12 |
| PBDB-BzT:IT-M | THF | 0.96 | 17.63 | 0.69 | 11.68 | S13 |
| PM7:IT-4F | Toluene | 0.88 | 20.9 | 71.1 | 13.1 | S14 |
| DRTT-T:F-2Cl | THF | 1.0 | 16.82 | 62.6 | 10.45 | S15 |
| P19:ITIC | Toluene | 0.938 | 15.68 | 68.03 | 10.01 | S16 |
| PTB-EDOTS:ITIC-Th | Me-THF | 0.88 | 16.25 | 71.2 | 10.18 | S17 |
| DOTFP-PhI :IT-4F | Toluene | 0.856 | 17.75 | 70.1 | 10.65 | S18 |
| P1:ITIC-Th | Toluene | 1.01 | 17.89 | 63.05 | 11.39 | S19 |
| PM6:IT-4F | *o*-xylene | 0.84 | 20.5 | 75.0 | 12.5 | S20 |
| P(Cl-Cl)(BDD=0.2):IT-4F | *o*-xylene | 0.891 | 23.4 | 67.0 | 13.97 | S21 |
| PTB7-Th:ACS8 | Toluene | 0.75 | 25.3 | 69.3 | 13.2 | S22 |
| T1:IT-4F | THF | 0.887 | 21.1 | 76.0 | 14.2 | S23 |
| T1:BTP-4F-12 | THF | 0.853 | 25.2 | 75.0 | 16.1 | S24 |
| PM6-Ir1.5:Y6-C2 | Toluene | 0.842 | 26.23 | 74.80 | 16.52 | S25 |
| PM6:DTY6 | *o*-xylene | 0.856 | 24.94 | 75.5 | 16.1 | S26 |
| PTQ10:HO-IDIC-2F | THF | 0.91 | 18.52 | 70.77 | 12.2 | S27 |
| PMT50:BTP-4F-12 | 1,2,4-TMB | 0.842 | 26.81 | 67.7 | 15.3 | S28 |
| PM6:BTP-BO-4Cl | Toluene | 0.854 | 26.1 | 77.7 | 17.33 | S29 |
| PTzBI-*d*F:CH1007 | *o*-xylene | 0.82 | 27.3 | 73 | 17.3 | S30 |
| PM6:YSe-C6 | *o*-xylene | 0.85 | 25.94 | 73.0 | 16.11 | S31 |

| | | | | | | |
|---|---|---|---|---|---|---|
| PM6:BTIC-2Cl-γ$CF_3$ | *o*-xylene | 0.84 | 25.09 | 76.99 | 16.31 | S32 |
| PTQ10:m-DTC-2Cl/PTB7-Th: BTPV-4F-eC9 | *o*-xylene/ THF | 1.621 | 14.65 | 70.2 | 16.67 | S33 |
| PM6:PM7:Y6:20% BTO | Paraxylene | 0.86 | 27.19 | 76.05 | 17.78 | S34 |
| PM6/BO-4F | *o*-xylene /THF | 0.82 | 26.2 | 74.3 | 16.0 | S35 |
| JD40-BDD20:PJTVT | *o*-xylene | 0.91 | 23.88 | 75.54 | 16.35 | S36 |
| PBQ6:PYF-T-*o*/DTT | Toluene | 0.886 | 25.12 | 76.64 | 17.06 | S37 |
| PM1:L8-BO:BTP-F3Cl | THF | 0.876 | 27.4 | 78.4 | 18.8 | S38 |
| PM6:10%5BDTBDD:BTP-BO4Cl | *o*-xylene | 0.843 | 26.83 | 77.43 | 17.54 | S39 |
| PL1:BTP-eC9-4F | *o*-xylene | 0.876 | 27.11 | 76.41 | 18.14 | S40 |
| PM6:PYTCl-A | Toluene | 0.939 | 21.71 | 72.12 | 14.7 | S41 |
| PBNT-TzTz:Y6-BO | Anisole | 0.88 | 25.42 | 70.0 | 15.65 | S42 |
| PM6: BTP-eC9-HD | Toluene | 0.83 | 26.6 | 78.8 | 17.5 | S43 |

**Table S4** Photovoltaic parameters of the OSCs based on active layers processed from spin-coating-free technologies

| Active layers | Solvent | $V_{OC}$ [V] | $J_{SC}$ [mA cm$^{-2}$] | FF | PCE [%] | Refs. |
|---|---|---|---|---|---|---|
| PM6:L8-BO:YBO-SeNF (1:1:0.2) | blade-coating | 0.869 | 27.3 | 0.776 | 18.4 | **This Work** |
| PM6:L8-BO:YBO-SeNF (1:0.8:0.4) | blade-coating | 0.861 | 27.5 | 0.772 | 18.2 | **This Work** |
| PM6:L8-BO:YHD-SeNF (1:1:0.2) | blade-coating | 0.874 | 27.3 | 0.789 | 18.8 | **This Work** |
| PM6:L8-BO:YHD-SeNF (1:0.8:0.4) | blade-coating | 0.866 | 28.0 | 0.775 | 18.8 | **This Work** |
| PM6:L8-BO:YDT-SeNF (1:1:0.2) | blade-coating | 0.871 | 26.9 | 0.788 | 18.5 | **This Work** |
| PM6:L8-BO:YDT-SeNF (1:0.8:0.4) | blade-coating | 0.864 | 27.8 | 0.775 | 18.6 | **This Work** |
| PCDTBT:PC71BM (20 $cm^2$) | slot-die | 0.88 | 10.66 | 55.2 | 5.18 | S44 |
| PffBT4T-2OD:PC71BM | blade-coating | 0.77 | 18.4 | 70 | 9.9 | S45 |
| PTB7-Th:PC70BM | slot-die | 2.36 | 4.98 | 62 | 8.1 | S46 |
| PBTA-TF:ITCC | blade-coating | 1.0 | 15.54 | 67 | 10.4 | S12 |
| PBTA-TF:IT-M | blade-coating | 0.97 | 17.76 | 71 | 12.2 | S12 |
| PBTA-TF:IT-4F | blade-coating | 0.73 | 20.24 | 72 | 10.6 | S12 |
| PBDB-T:IT-M | blade-coating | 0.73 | 24.61 | 65.51 | 12.02 | S47 |
| P3HT:IDTBR (60 $cm^2$) | slot-die | 0.71 | 1.08 | 64.9 | 5.0 | S48 |
| PBDB-T-2F:IT-4F | blade-coating | 0.86 | 21.3 | 76 | 13.9 | S49 |
| PBDB-T:ITIC | blade-coating | 0.9 | 17.4 | 69 | 10.9 | S49 |
| PBDB-T:IT-M | blade-coating | 0.94 | 16.9 | 71 | 11.3 | S49 |

| | | | | | | |
|---|---|---|---|---|---|---|
| PBDB-T:IDIC | blade-coating | 0.98 | 15.5 | 60 | 9.1 | S49 |
| PM6:IT-4F | blade-coating | 0.82 | 18.9 | 64.0 | 9.9 | S50 |
| PM6:IT-4F:N2200 | blade-coating | 0.86 | 20.8 | 73.0 | 12.8 | S50 |
| PM6:Y6 | blade-coating | 0.81 | 24.4 | 66.0 | 12.6 | S50 |
| PM6:Y6:N2200 | blade-coating | 0.83 | 26.3 | 74.0 | 15.6 | S50 |
| PTB7-Th:$PC_{71}$BM | blade-coating | 0.79 | 16.39 | 69.0 | 9.33 | S51 |
| PBDB-TF:IT-4F | blade-coating | 0.86 | 20.8 | 72.0 | 12.88 | S51 |
| PM6:IT-4F | blade-coating | 0.88 | 20.76 | 72.9 | 13.64 | S52 |
| PBDB-T:IT-M | blade-coating | 0.93 | 16.8 | 76.0 | 11.8 | S53 |
| PNTz4T-5MTC:PC71BM (54.45 $cm^2$) | slot-die | 7.97 | 1.34 | 61.0 | 6.61 | S54 |
| PBDTTT-EFT:PC71BM | blade-coating | 0.82 | 19.44 | 63.0 | 9.75 | S55 |
| PBDTTT-EFT:PC71BM | blade-coating | 0.85 | 17.39 | 67.0 | 9.39 | S55 |
| PBDTTT-EFT:PC71BM | blade-coating | 0.82 | 17.89 | 74.0 | 11.09 | S55 |
| J71:ITC6-IC | blade-coating | 0.96 | 17.61 | 67.54 | 11.42 | S56 |
| PTQ10:IDIC | blade-coating | 0.952 | 17.14 | 69.13 | 11.28 | S56 |
| PBDB-T-SF:IT-4F | slot-die | 0.87 | 20.13 | 70.11 | 12.3 | S57 |
| J71:ITC6-IC | blade-coating | 0.968 | 16.85 | 70.1 | 11.47 | S58 |
| PCE10:Y8 | blade-coating | 0.703 | 20.53 | 60.65 | 8.75 | S59 |
| PCE10:Y8:ICBA | blade-coating | 0.722 | 20.97 | 62.29 | 9.52 | S59 |
| PTB7-Th:F10IC2 | blade-coating | 0.766 | 23.8 | 68.5 | 12.5 | S60 |
| PBDB-TF:BTP-4Cl-8 | blade-coating | 0.838 | 21.7 | 63.5 | 11.5 | S61 |
| PBDB-TF:BTP-4Cl-12 | blade-coating | 0.833 | 26.0 | 71.6 | 15.5 | S61 |
| PBDB-TF:BTP-4Cl-16 | blade-coating | 0.807 | 19.4 | 68.9 | 10.8 | S61 |
| PT2:Y6 | blade-coating | 0.83 | 26.3 | 68.9 | 15.0 | S62 |
| PM6:Y6 | blade-coating | 0.834 | 25.9 | 75.68 | 16.35 | S63 |
| PM6:Y6-2Cl | blade-coating | 0.849 | 25.88 | 72.3 | 15.89 | S63 |
| PTQ10:Y6 | blade-coating | 0.849 | 24.49 | 72.63 | 15.1 | S63 |
| PM6:Y6-C2 | blade-coating | 0.834 | 25.82 | 73.99 | 15.93 | S63 |
| PBDB-T:FOIC:IT-M | blade-coating | 0.751 | 24.66 | 63.57 | 11.86 | S64 |
| PM6:Y6 | blade-coating | 0.834 | 24.9 | 74.6 | 15.51 | S65 |
| PM6:Y6 | blade-coating | 0.833 | 26.25 | 68.86 | 15.5 | S66 |
| PM6:BTP-eC9 | blade-coating | 0.839 | 25.87 | 77.0 | 16.71 | S67 |

| | | | | | | |
|---|---|---|---|---|---|---|
| D18:BTR-Cl:Y6 | slot-die | 0.85 | 27.9 | 68.5 | 16.3 | S68 |
| PTQ10:Y6 | blade-coating | 0.842 | 25.6 | 67.1 | 14.5 | S69 |
| PM6:L8-BO (LBL) | slot-die | 0.867 | 25.37 | 77.23 | 16.95 | S70 |
| PM6:T8 (BHJ) | slot-die | 0.860 | 25.89 | 72.09 | 16.05 | S71 |
| PM6:T8 (LBL) | slot-die | 0.866 | 26.21 | 74.29 | 16.86 | S71 |
| PM6:T8 (BHJ) | blade-coating | 0.866 | 26.2 | 76.93 | 17.45 | S71 |
| PM6/T8 (LBL) | blade-coating | 0.864 | 26.98 | 77.13 | 17.98 | S71 |

**Table S5** Photovoltaic data of the 20 independent OSCs based on PM6:L8-BO

| | $V_{OC}$ [V] | $J_{SC}$ [mA cm$^{-2}$] | FF | PCE [%] | | $V_{OC}$ [V] | $J_{SC}$ [mA cm$^{-2}$] | FF | PCE [%] |
|---|---|---|---|---|---|---|---|---|---|
| No.1 | 0.880 | 25.3 | 0.779 | 17.3 | No.11 | 0.874 | 25.6 | 0.772 | 17.3 |
| No.2 | 0.881 | 25.0 | 0.778 | 17.1 | No.12 | 0.883 | 24.8 | 0.775 | 17.0 |
| No.3 | 0.875 | 25.2 | 0.771 | 17.0 | No.13 | 0.880 | 25.2 | 0.778 | 17.3 |
| No.4 | 0.872 | 25.6 | 0.766 | 17.1 | No.14 | 0.877 | 25.4 | 0.775 | 17.3 |
| No.5 | 0.868 | 25.7 | 0.763 | 17.0 | No.15 | 0.876 | 25.5 | 0.771 | 17.2 |
| No.6 | 0.878 | 24.8 | 0.781 | 17.0 | No.16 | 0.874 | 25.7 | 0.769 | 17.3 |
| No.7 | 0.877 | 24.6 | 0.775 | 16.7 | No.17 | 0.872 | 25.1 | 0.766 | 16.8 |
| No.8 | 0.869 | 25.5 | 0.761 | 16.9 | No.18 | 0.867 | 25.9 | 0.758 | 17.0 |
| No.9 | 0.882 | 24.3 | 0.782 | 16.8 | No.19 | 0.873 | 25.2 | 0.775 | 17.0 |
| No.10 | 0.883 | 24.2 | 0.781 | 16.7 | No.20 | 0.879 | 25 | 0.777 | 17.1 |

**Table S6** Photovoltaic data of the 20 independent OSCs based on PM6:L8-BO:YBO-SeNF (1:1:0.2)

| | $V_{OC}$ [V] | $J_{SC}$ [mA cm$^{-2}$] | FF | PCE [%] | | $V_{OC}$ [V] | $J_{SC}$ [mA cm$^{-2}$] | FF | PCE [%] |
|---|---|---|---|---|---|---|---|---|---|
| No.1 | 0.869 | 27.3 | 0.776 | 18.4 | No.11 | 0.868 | 27.2 | 0.775 | 18.3 |
| No.2 | 0.865 | 27.1 | 0.772 | 18.1 | No.12 | 0.863 | 26.7 | 0.765 | 17.6 |
| No.3 | 0.859 | 27.5 | 0.768 | 18.1 | No.13 | 0.862 | 26.8 | 0.758 | 17.5 |
| No.4 | 0.855 | 26.8 | 0.778 | 17.8 | No.14 | 0.868 | 27.5 | 0.765 | 18.3 |
| No.5 | 0.862 | 27.2 | 0.771 | 18.1 | No.15 | 0.868 | 27.2 | 0.777 | 18.3 |
| No.6 | 0.863 | 27.1 | 0.766 | 17.9 | No.16 | 0.869 | 26.8 | 0.78 | 18.2 |
| No.7 | 0.861 | 26.7 | 0.781 | 18.0 | No.17 | 0.872 | 26.2 | 0.782 | 17.9 |

| | | | | | | | | | |
|---|---|---|---|---|---|---|---|---|---|
| No.8 | 0.872 | 26.1 | 0.782 | 17.8 | No.18 | 0.867 | 27.1 | 0.778 | 18.3 |
| No.9 | 0.861 | 27 | 0.775 | 18.0 | No.19 | 0.865 | 27 | 0.775 | 18.1 |
| No.10 | 0.87 | 26.8 | 0.772 | 18.0 | No.20 | 0.866 | 26.5 | 0.778 | 17.9 |

**Table S7** Photovoltaic data of the 20 independent OSCs based on PM6:L8-BO:YBO-SeNF (1:0.8:0.4)

| | $V_{OC}$ [V] | $J_{SC}$ [mA cm$^{-2}$] | FF | PCE [%] | | $V_{OC}$ [V] | $J_{SC}$ [mA cm$^{-2}$] | FF | PCE [%] |
|---|---|---|---|---|---|---|---|---|---|
| No.1 | 0.861 | 27.5 | 0.772 | 18.2 | No.11 | 0.854 | 27.6 | 0.759 | 17.9 |
| No.2 | 0.862 | 27 | 0.775 | 18.0 | No.12 | 0.860 | 27.4 | 0.774 | 18.2 |
| No.3 | 0.860 | 27.6 | 0.765 | 18.2 | No.13 | 0.861 | 27.3 | 0.77 | 18.1 |
| No.4 | 0.855 | 27.3 | 0.758 | 17.7 | No.14 | 0.862 | 27.1 | 0.769 | 18.0 |
| No.5 | 0.856 | 27.2 | 0.761 | 17.7 | No.15 | 0.859 | 27.7 | 0.766 | 18.2 |
| No.6 | 0.851 | 27.8 | 0.755 | 17.9 | No.16 | 0.858 | 27.5 | 0.772 | 18.2 |
| No.7 | 0.852 | 27.6 | 0.766 | 18.0 | No.17 | 0.860 | 27.1 | 0.771 | 18.0 |
| No.8 | 0.856 | 27.2 | 0.768 | 17.9 | No.18 | 0.853 | 27.6 | 0.763 | 18.0 |
| No.9 | 0.852 | 26.8 | 0.771 | 17.6 | No.19 | 0.852 | 27.1 | 0.77 | 17.8 |
| No.10 | 0.862 | 26.3 | 0.773 | 17.5 | No.20 | 0.857 | 26.9 | 0.768 | 17.7 |

**Table S8** Photovoltaic data of the 20 independent OSCs based on PM6:YBO-SeNF

| | $V_{OC}$ [V] | $J_{SC}$ [mA cm$^{-2}$] | FF | PCE [%] | | $V_{OC}$ [V] | $J_{SC}$ [mA cm$^{-2}$] | FF | PCE [%] |
|---|---|---|---|---|---|---|---|---|---|
| No.1 | 0.824 | 27.1 | 0.745 | 16.6 | No.11 | 0.820 | 26.5 | 0.740 | 16.1 |
| No.2 | 0.818 | 27.3 | 0.728 | 16.3 | No.12 | 0.817 | 27 | 0.732 | 16.1 |
| No.3 | 0.811 | 27.5 | 0.719 | 16.0 | No.13 | 0.827 | 26.6 | 0.742 | 16.3 |
| No.4 | 0.825 | 26.5 | 0.742 | 16.2 | No.14 | 0.822 | 26.9 | 0.735 | 16.3 |
| No.5 | 0.828 | 26.3 | 0.746 | 16.2 | No.15 | 0.819 | 27.5 | 0.711 | 16.0 |
| No.6 | 0.829 | 25.9 | 0.741 | 15.9 | No.16 | 0.813 | 27.6 | 0.714 | 16.0 |
| No.7 | 0.812 | 27.2 | 0.716 | 15.8 | No.17 | 0.825 | 27 | 0.742 | 16.5 |
| No.8 | 0.816 | 26.5 | 0.725 | 15.7 | No.18 | 0.829 | 26 | 0.751 | 16.2 |
| No.9 | 0.823 | 26.7 | 0.737 | 16.2 | No.19 | 0.824 | 26.7 | 0.747 | 16.4 |
| No.10 | 0.822 | 26.9 | 0.746 | 16.5 | No.20 | 0.821 | 26.5 | 0.732 | 15.9 |

**Table S9** Photovoltaic data of the 20 independent OSCs based on PM6:L8-BO:YHD-SeNF (1:1:0.2)

| | $V_{OC}$ [V] | $J_{SC}$ [mA cm$^{-2}$] | FF | PCE [%] | | $V_{OC}$ [V] | $J_{SC}$ [mA cm$^{-2}$] | FF | PCE [%] |
|---|---|---|---|---|---|---|---|---|---|
| No.1 | 0.874 | 27.3 | 0.789 | 18.8 | No.11 | 0.871 | 26.8 | 0.785 | 18.3 |
| No.2 | 0.871 | 27.1 | 0.785 | 18.5 | No.12 | 0.88 | 26.1 | 0.793 | 18.2 |
| No.3 | 0.867 | 27.5 | 0.776 | 18.5 | No.13 | 0.873 | 27.2 | 0.786 | 18.7 |
| No.4 | 0.878 | 26.2 | 0.792 | 18.2 | No.14 | 0.874 | 27.2 | 0.788 | 18.7 |
| No.5 | 0.870 | 27.6 | 0.781 | 18.8 | No.15 | 0.875 | 27.0 | 0.782 | 18.5 |
| No.6 | 0.868 | 27.5 | 0.78 | 18.6 | No.16 | 0.879 | 26.4 | 0.788 | 18.3 |
| No.7 | 0.859 | 27.2 | 0.776 | 18.1 | No.17 | 0.872 | 27.1 | 0.782 | 18.5 |
| No.8 | 0.862 | 27.6 | 0.772 | 18.4 | No.18 | 0.871 | 27.0 | 0.777 | 18.3 |
| No.9 | 0.871 | 26.6 | 0.776 | 18.0 | No.19 | 0.874 | 27.3 | 0.783 | 18.7 |
| No.10 | 0.873 | 27.0 | 0.772 | 18.2 | No.20 | 0.875 | 27.2 | 0.781 | 18.6 |

**Table S10** Photovoltaic data of the 20 independent OSCs based on PM6:L8-BO:YHD-SeNF (1:0.8:0.4)

| | $V_{OC}$ [V] | $J_{SC}$ [mA cm$^{-2}$] | FF | PCE [%] | | $V_{OC}$ [V] | $J_{SC}$ [mA cm$^{-2}$] | FF | PCE [%] |
|---|---|---|---|---|---|---|---|---|---|
| No.1 | 0.866 | 28.0 | 0.775 | 18.8 | No.11 | 0.852 | 28.1 | 0.759 | 18.2 |
| No.2 | 0.862 | 28.2 | 0.766 | 18.6 | No.12 | 0.851 | 27.9 | 0.763 | 18.1 |
| No.3 | 0.86 | 28.0 | 0.763 | 18.4 | No.13 | 0.857 | 27.5 | 0.77 | 18.1 |
| No.4 | 0.852 | 28.5 | 0.758 | 18.4 | No.14 | 0.865 | 27.1 | 0.772 | 18.1 |
| No.5 | 0.855 | 28.1 | 0.763 | 18.3 | No.15 | 0.866 | 27.2 | 0.778 | 18.3 |
| No.6 | 0.868 | 27.6 | 0.776 | 18.6 | No.16 | 0.861 | 28.3 | 0.761 | 18.5 |
| No.7 | 0.867 | 27.2 | 0.779 | 18.4 | No.17 | 0.865 | 28.1 | 0.773 | 18.8 |
| No.8 | 0.861 | 27.9 | 0.775 | 18.6 | No.18 | 0.853 | 27.5 | 0.758 | 17.8 |
| No.9 | 0.857 | 28.3 | 0.773 | 18.7 | No.19 | 0.849 | 28.8 | 0.745 | 18.2 |
| No.10 | 0.855 | 27.7 | 0.769 | 18.2 | No.20 | 0.869 | 26.9 | 0.776 | 18.1 |

**Table S11** Photovoltaic data of the 20 independent OSCs based on PM6:YHD-SeNF

| | $V_{OC}$ [V] | $J_{SC}$ [mA cm$^{-2}$] | FF | PCE [%] | | $V_{OC}$ [V] | $J_{SC}$ [mA cm$^{-2}$] | FF | PCE [%] |
|---|---|---|---|---|---|---|---|---|---|
| No.1 | 0.848 | 26.4 | 0.753 | 16.8 | No.11 | 0.844 | 25.9 | 0.760 | 16.6 |
| No.2 | 0.853 | 25.5 | 0.755 | 16.4 | No.12 | 0.84 | 25.7 | 0.739 | 16.0 |
| No.3 | 0.847 | 26.2 | 0.750 | 16.6 | No.13 | 0.826 | 26.6 | 0.722 | 15.9 |

| | | | | | | | | | |
|---|---|---|---|---|---|---|---|---|---|
| No.4 | 0.842 | 26.5 | 0.749 | 16.7 | No.14 | 0.839 | 25.5 | 0.734 | 15.7 |
| No.5 | 0.843 | 26.0 | 0.742 | 16.3 | No.15 | 0.842 | 25.3 | 0.758 | 16.1 |
| No.6 | 0.837 | 26.3 | 0.749 | 16.5 | No.16 | 0.832 | 26.1 | 0.748 | 16.2 |
| No.7 | 0.835 | 25.8 | 0.752 | 16.2 | No.17 | 0.837 | 26.2 | 0.741 | 16.2 |
| No.8 | 0.831 | 26.7 | 0.733 | 16.3 | No.18 | 0.833 | 25.9 | 0.739 | 15.9 |
| No.9 | 0.841 | 25.8 | 0.729 | 15.8 | No.19 | 0.841 | 25.2 | 0.756 | 16.0 |
| No.10 | 0.828 | 26.5 | 0.736 | 16.1 | No.20 | 0.849 | 26.1 | 0.751 | 16.6 |

**Table S12** Photovoltaic data of the 20 independent OSCs based on PM6:L8-BO:YDT-SeNF (1:1:0.2)

| | $V_{OC}$ [V] | $J_{SC}$ [mA cm$^{-2}$] | FF | PCE [%] | | $V_{OC}$ [V] | $J_{SC}$ [mA cm$^{-2}$] | FF | PCE [%] |
|---|---|---|---|---|---|---|---|---|---|
| No.1 | 0.871 | 26.9 | 0.788 | 18.5 | No.11 | 0.856 | 27.4 | 0.770 | 18.1 |
| No.2 | 0.865 | 26.5 | 0.785 | 18.0 | No.12 | 0.869 | 26.2 | 0.784 | 17.8 |
| No.3 | 0.866 | 27.0 | 0.781 | 18.3 | No.13 | 0.862 | 27.1 | 0.787 | 18.4 |
| No.4 | 0.860 | 27.2 | 0.773 | 18.1 | No.14 | 0.875 | 25.9 | 0.795 | 18.0 |
| No.5 | 0.872 | 26.3 | 0.791 | 18.1 | No.15 | 0.869 | 27.2 | 0.780 | 18.4 |
| No.6 | 0.857 | 27.1 | 0.772 | 17.9 | No.16 | 0.861 | 27.0 | 0.778 | 18.1 |
| No.7 | 0.858 | 26.8 | 0.783 | 18.0 | No.17 | 0.865 | 26.2 | 0.783 | 17.7 |
| No.8 | 0.864 | 27.0 | 0.769 | 17.9 | No.18 | 0.870 | 26.5 | 0.784 | 18.1 |
| No.9 | 0.873 | 26.3 | 0.774 | 17.8 | No.19 | 0.862 | 26.9 | 0.779 | 18.1 |
| No.10 | 0.870 | 26.5 | 0.791 | 18.2 | No.20 | 0.855 | 27.2 | 0.782 | 18.2 |

**Table S13** Photovoltaic data of the 20 independent OSCs based on PM6:L8-BO:YDT-SeNF (1:0.8:0.4)

| | $V_{OC}$ [V] | $J_{SC}$ [mA cm$^{-2}$] | FF | PCE [%] | | $V_{OC}$ [V] | $J_{SC}$ [mA cm$^{-2}$] | FF | PCE [%] |
|---|---|---|---|---|---|---|---|---|---|
| No.1 | 0.864 | 27.8 | 0.775 | 18.6 | No.11 | 0.847 | 28.2 | 0.762 | 18.2 |
| No.2 | 0.860 | 28.0 | 0.771 | 18.6 | No.12 | 0.858 | 27.6 | 0.777 | 18.4 |
| No.3 | 0.852 | 27.6 | 0.762 | 17.9 | No.13 | 0.856 | 27.9 | 0.770 | 18.4 |
| No.4 | 0.856 | 27.1 | 0.769 | 17.8 | No.14 | 0.867 | 26.6 | 0.780 | 18.0 |
| No.5 | 0.852 | 27.5 | 0.772 | 18.1 | No.15 | 0.863 | 27.2 | 0.782 | 18.4 |
| No.6 | 0.857 | 27.5 | 0.776 | 18.3 | No.16 | 0.865 | 27.5 | 0.771 | 18.3 |
| No.7 | 0.866 | 26.9 | 0.766 | 17.8 | No.17 | 0.866 | 27.1 | 0.780 | 18.3 |
| No.8 | 0.867 | 27.2 | 0.778 | 18.3 | No.18 | 0.853 | 28.2 | 0.767 | 18.4 |

| | | | | | | | | | |
|---|---|---|---|---|---|---|---|---|---|
| No.9 | 0.863 | 27.9 | 0.772 | 18.6 | No.19 | 0.852 | 27.5 | 0.764 | 17.9 |
| No.10 | 0.848 | 28.1 | 0.769 | 18.3 | No.20 | 0.861 | 27.7 | 0.773 | 18.4 |

**Table S14** Photovoltaic data of the 20 independent OSCs based on PM6:YDT-SeNF

| | $V_{OC}$ [V] | $J_{SC}$ [mA cm$^{-2}$] | FF | PCE [%] | | $V_{OC}$ [V] | $J_{SC}$ [mA cm$^{-2}$] | FF | PCE [%] |
|---|---|---|---|---|---|---|---|---|---|
| No.1 | 0.836 | 27.2 | 0.754 | 17.2 | No.11 | 0.838 | 26.7 | 0.759 | 17.0 |
| No.2 | 0.828 | 27.3 | 0.746 | 16.9 | No.12 | 0.833 | 27.5 | 0.744 | 17.0 |
| No.3 | 0.823 | 27.6 | 0.739 | 16.8 | No.13 | 0.819 | 27.7 | 0.729 | 16.5 |
| No.4 | 0.837 | 26.1 | 0.761 | 16.6 | No.14 | 0.818 | 27.8 | 0.736 | 16.7 |
| No.5 | 0.836 | 26.3 | 0.755 | 16.6 | No.15 | 0.822 | 27.6 | 0.730 | 16.6 |
| No.6 | 0.834 | 27.0 | 0.755 | 17.0 | No.16 | 0.827 | 27.0 | 0.744 | 16.6 |
| No.7 | 0.827 | 26.9 | 0.743 | 16.5 | No.17 | 0.837 | 26.7 | 0.755 | 16.9 |
| No.8 | 0.829 | 27.1 | 0.751 | 16.9 | No.18 | 0.841 | 26.5 | 0.753 | 16.8 |
| No.9 | 0.834 | 26.7 | 0.754 | 16.8 | No.19 | 0.831 | 27.7 | 0.732 | 16.8 |
| No.10 | 0.840 | 26.3 | 0.761 | 16.8 | No.20 | 0.825 | 28.0 | 0.726 | 16.8 |

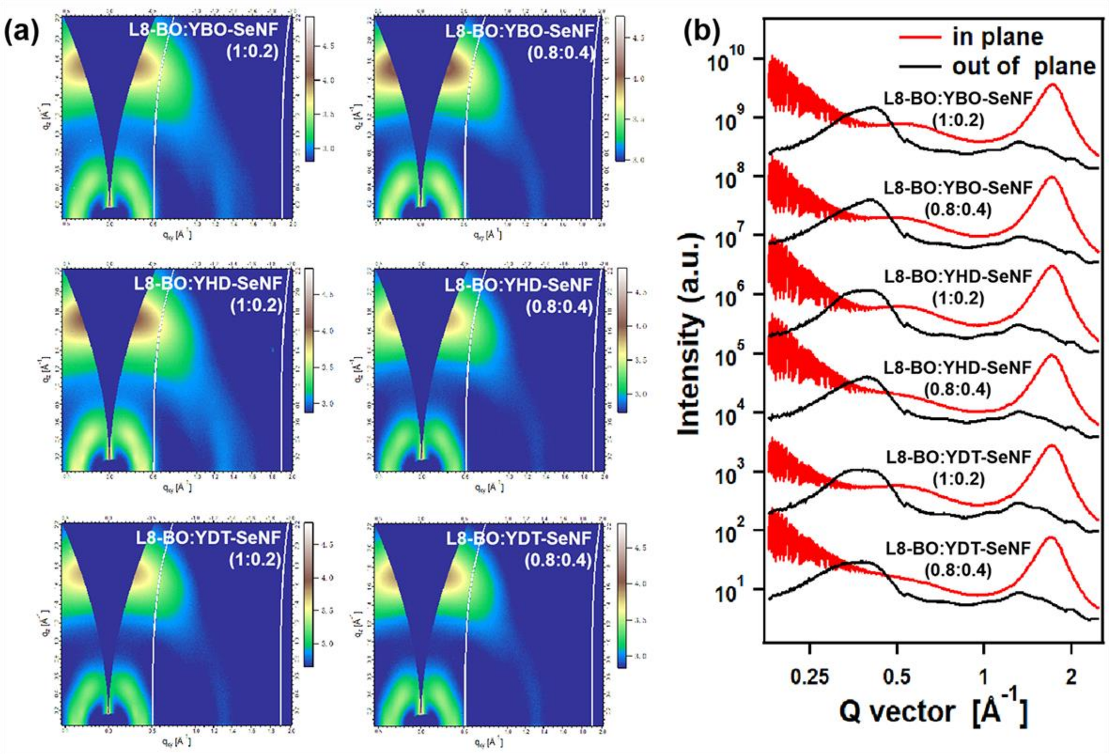


**Fig. S22** (**a**) GIWAXS profiles of blade-coating L8-BO:YR-SeNF (1:0.2 and 0.8:0.4) binary blends and (**b**) the corresponding line-cuts

**Table S15** GIWAXS test performance parameters of L8-BO:YR-SeNF binary blends

| | in plane | | | | | |
|---|---|---|---|---|---|---|
| | location (Å$^{-1}$) | d-spacing (Å) | CCL (Å) | location (Å$^{-1}$) | d-spacing (Å) | CCL (Å) |
| L8-BO:YBO-SeNF (1:0.2) | 0.341 | 18.438 | 49.181 | 0.425 | 14.783 | 54.160 |
| L8-BO:YBO-SeNF (0.8:0.4) | 0.343 | 18.308 | 40.467 | 0.419 | 15.007 | 56.273 |
| L8-BO:YHD-SeNF (1:0.2) | 0.341 | 18.399 | 50.798 | 0.425 | 14.785 | 45.766 |
| L8-BO:YHD-SeNF (0.8:0.4) | 0.336 | 18.715 | 44.962 | 0.412 | 15.247 | 55.083 |
| L8-BO:YDT-SeNF (1:0.2) | 0.352 | 17.858 | 46.083 | 0.432 | 14.532 | 67.185 |
| L8-BO:YDT-SeNF (0.8:0.4) | 0.319 | 19.671 | 45.655 | 0.409 | 15.345 | 51.516 |
| | out of plane | | | | | |
| | location (Å$^{-1}$) | d-spacing (Å) | CCL (Å) | location (Å$^{-1}$) | d-spacing (Å) | CCL (Å) |
| L8-BO:YBO-SeNF (1:0.2) | 1.721 | 3.651 | 16.247 | | | |
| L8-BO:YBO-SeNF (0.8:0.4) | 1.712 | 3.671 | 16.487 | | | |
| L8-BO:YHD-SeNF (1:0.2) | 1.713 | 3.667 | 16.502 | | | |
| L8-BO:YHD-SeNF (0.8:0.4) | 1.710 | 3.675 | 16.288 | | | |
| L8-BO:YDT-SeNF (1:0.2) | 1.710 | 3.674 | 16.023 | | | |
| L8-BO:YDT-SeNF (0.8:0.4) | 1.700 | 3.697 | 16.009 | | | |

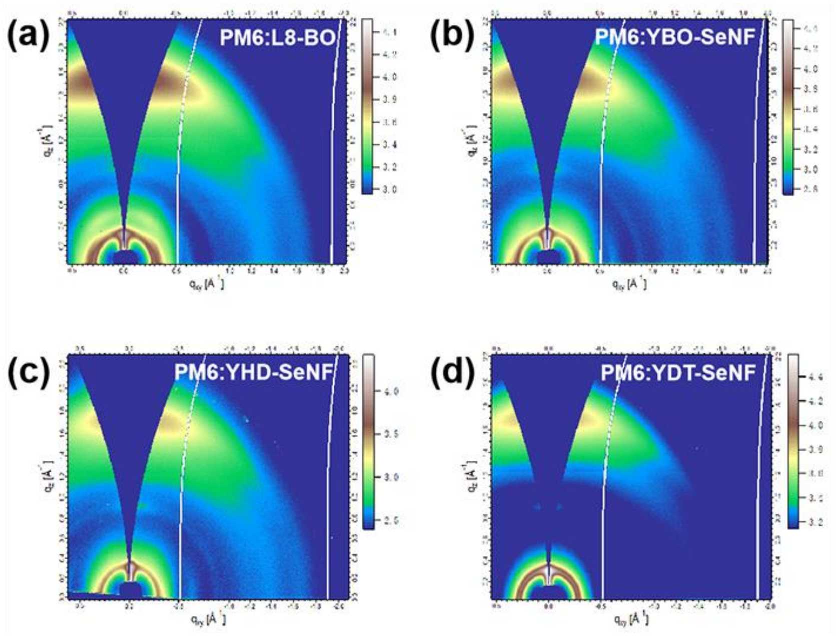


**Fig. S23** GIWAXS profiles of blade-coating binary blends of (**a**) PM6:L8-BO, (**b**) PM6:YBO-SeNF, (**c**) PM6:YHD-SeNF, and (**d**) PM6:YDT-SeNF

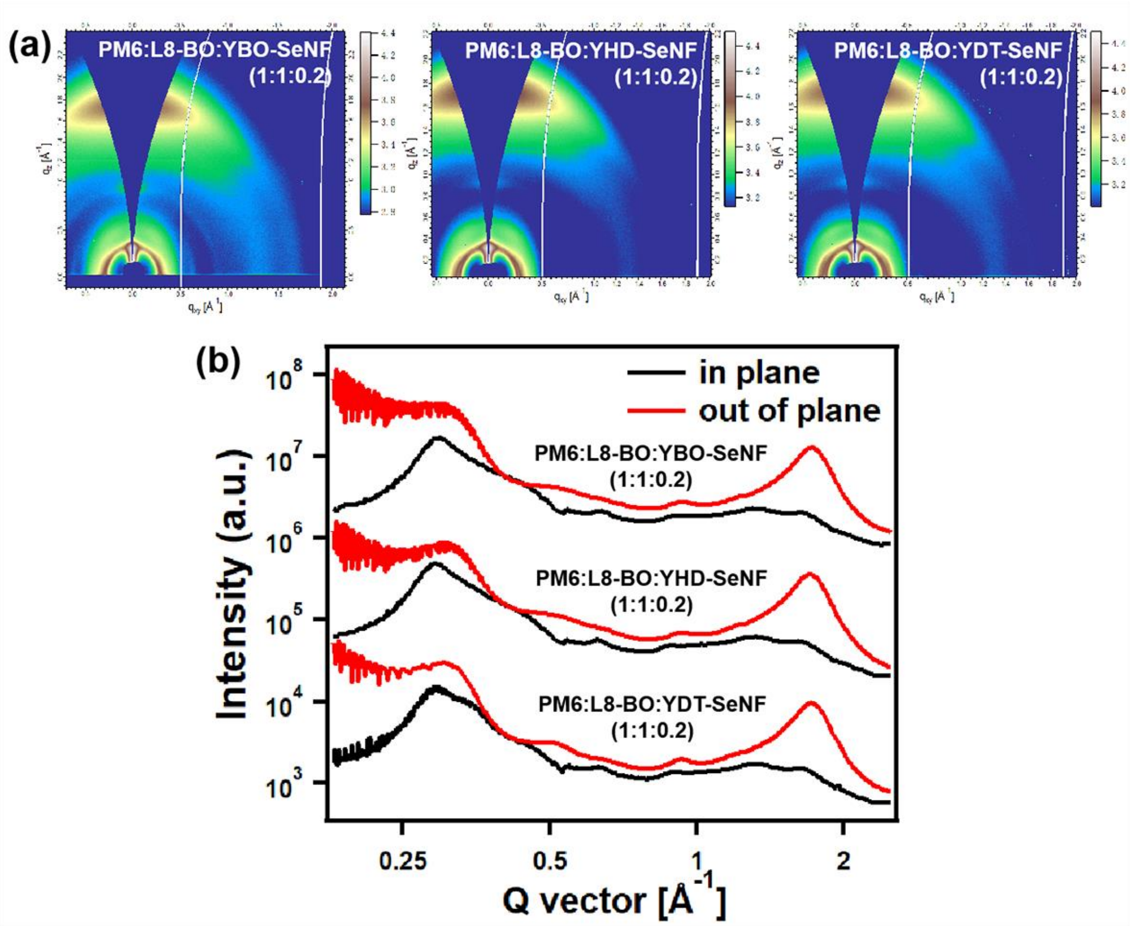


**Fig. S24** (**a**) GIWAXS profiles of blade-coating PM6:L8-BO:YR-SeNF (1:1:0.2) blends and (**b**) the corresponding line-cuts

**Table S16** GIWAXS test performance parameters of PM6:L8-BO:YR-SeNF blends with different component ratios

| | in plane | | | | | |
|---|---|---|---|---|---|---|
| | location (Å$^{-1}$) | d-spacing (Å) | CCL (Å) | location (Å$^{-1}$) | d-spacing (Å) | CCL (Å) |
| PM6:L8-BO:YBO-SeNF (1:1.2:0) | 0.293 | 21.426 | 94.764 | 0.348 | 18.081 | 111.04 |
| | 0.403 | 15.608 | 52.326 | | | |
| PM6:L8-BO:YBO-SeNF (1:1:0.2) | 0.297 | 21.151 | 91.900 | 0.354 | 17.766 | 129.91 |
| | 0.398 | 15.780 | 56.374 | | | |
| PM6:L8-BO:YBO-SeNF (1:0.8:0.4) | 0.296 | 21.259 | 93.676 | 0.352 | 17.834 | 136.26 |
| | 0.395 | 15.890 | 69.113 | | | |
| PM6:L8-BO:YBO-SeNF (1:0:1.2) | 0.293 | 21.470 | 101.12 | 0.361 | 17.405 | 56.603 |
| PM6:L8-BO:YHD-SeNF (1:1:0.2) | 0.291 | 21.618 | 114.10 | 0.347 | 18.109 | 124.41 |
| | 0.394 | 15.941 | 52.889 | | | |
| PM6:L8-BO:YHD-SeNF (1:0.8:0.4) | 0.294 | 21.379 | 96.664 | 0.348 | 18.058 | 127.55 |
| | 0.392 | 16.044 | 61.455 | | | |
| PM6:L8-BO:YHD-SeNF (1:0:1.2) | 0.293 | 21.479 | 94.414 | 0.354 | 17.750 | 66.232 |
| PM6:L8-BO:YDT-SeNF (1:1:0.2) | 0.292 | 21.514 | 98.263 | 0.345 | 18.217 | 113.12 |
| | 0.402 | 15.613 | 60.009 | | | |
| PM6:L8-BO:YDT-SeNF (1:0.8:0.4) | 0.294 | 21.348 | 81.951 | 0.358 | 17.561 | 120.02 |
| | 0.402 | 15.610 | 52.574 | | | |
| PM6:L8-BO:YDT-SeNF (1:0:1.2) | 0.285 | 22.045 | 87.276 | | | |
| | out of plane | | | | | |
| | location (Å$^{-1}$) | d-spacing (Å) | CCL (Å) | location (Å$^{-1}$) | d-spacing (Å) | CCL (Å) |
| PM6:L8-BO:YBO-SeNF (1:1.2:0) | 1.709 | 3.677 | 17.231 | | | |
| PM6:L8-BO:YBO-SeNF (1:1:0.2) | 1.713 | 3.668 | 17.863 | | | |
| PM6:L8-BO:YBO-SeNF (1:0.8:0.4) | 1.709 | 3.676 | 17.995 | | | |
| PM6:L8-BO:YBO-SeNF (1:0:1.2) | 1.706 | 3.684 | 16.963 | | | |
| PM6:L8-BO:YHD-SeNF | 1.697 | 3.702 | 16.918 | | | |

| | | | | |
|---|---|---|---|---|
| (1:1:0.2) | | | | |
| PM6:L8-BO:YHD-SeNF (1:0.8:0.4) | 1.695 | 3.705 | 16.628 | |
| PM6:L8-BO:YHD-SeNF (1:0:1.2) | 1.690 | 3.717 | 16.359 | |
| PM6:L8-BO:YDT-SeNF (1:1:0.2) | 1.707 | 3.681 | 17.126 | |
| PM6:L8-BO:YDT-SeNF (1:0.8:0.4) | 1.690 | 3.718 | 16.432 | |
| PM6:L8-BO:YDT-SeNF (1:0:1.2) | 1.676 | 3.749 | 15.361 | |

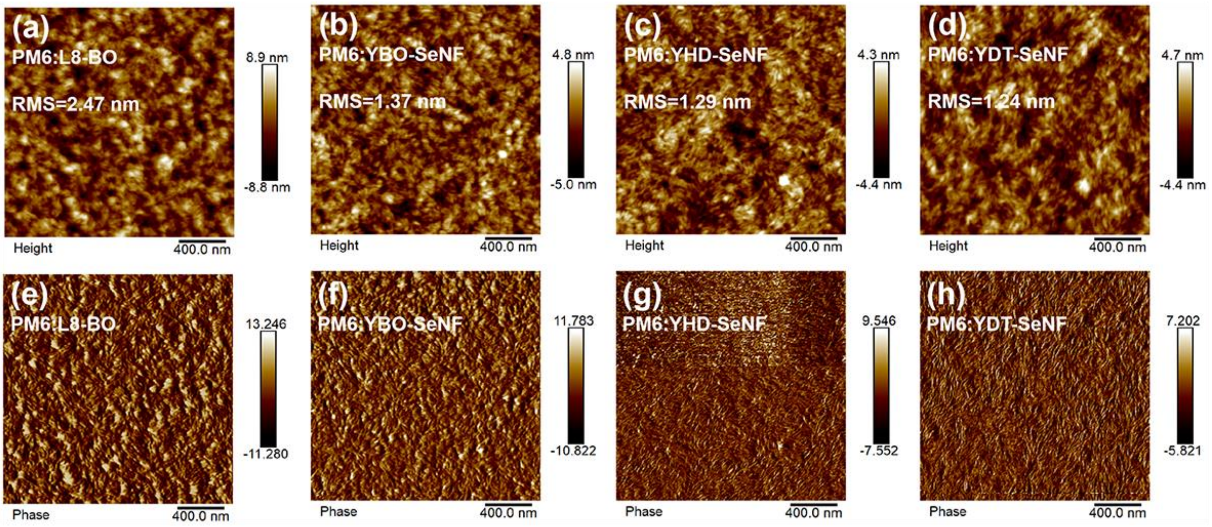


**Fig. S25** AFM height images (a-d) and phase images (e-h) of PM6:L8-BO and PM6:YR-SeNF binary blends

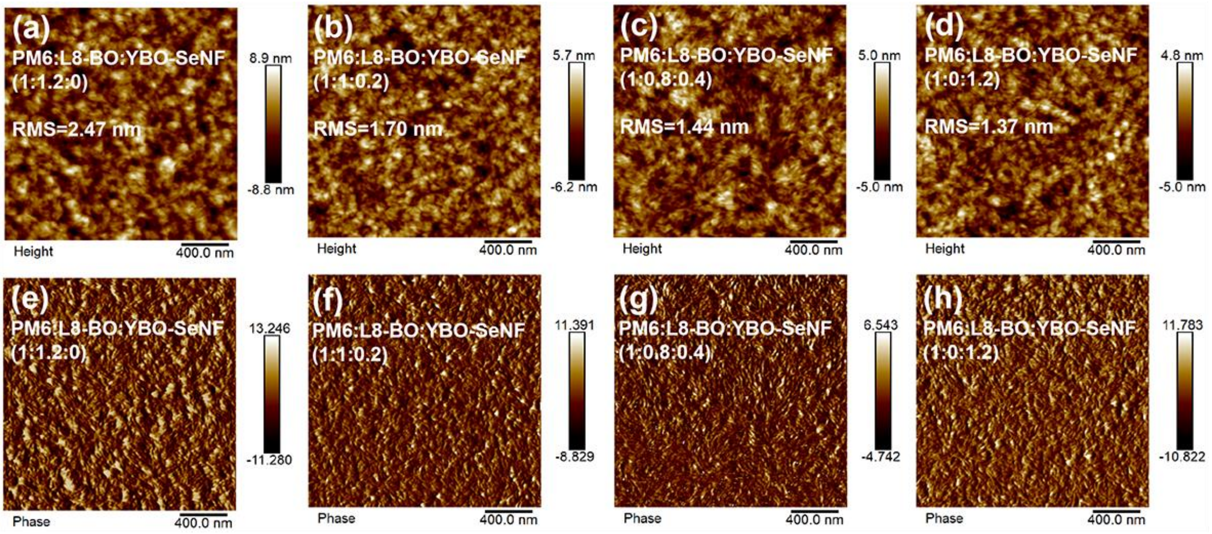


**Fig. S26** AFM height images (**a-d**) and phase images (**e-h**) of PM6:L8-BO:YBO-SeNF blends with different component ratios

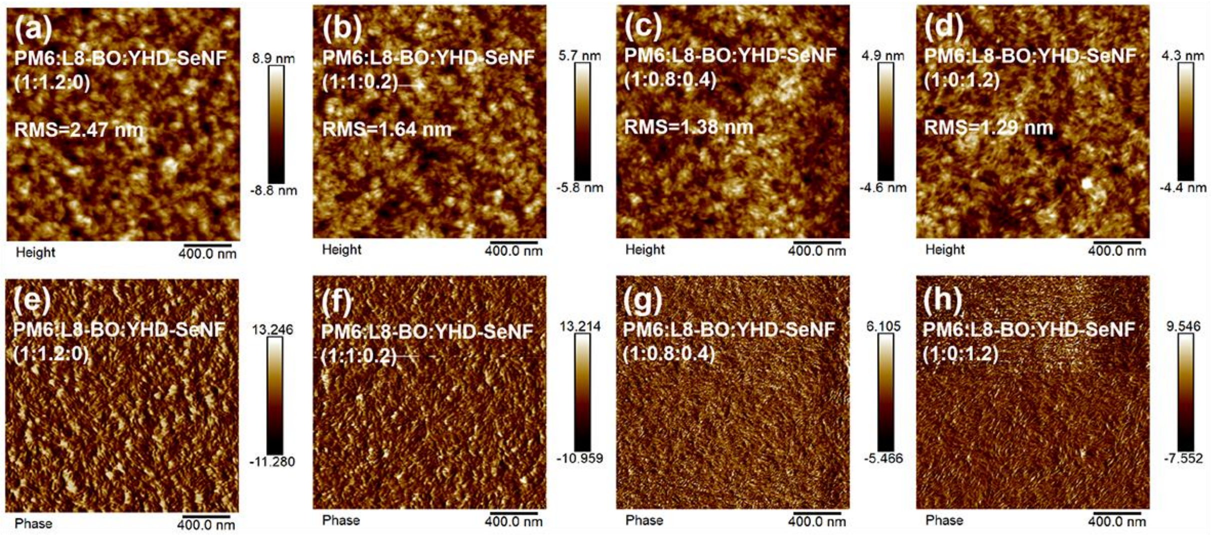


**Fig. S27** AFM height images (**a-d**) and phase images (**e-h**) of PM6:L8-BO:YHD-SeNF blends with different component ratios

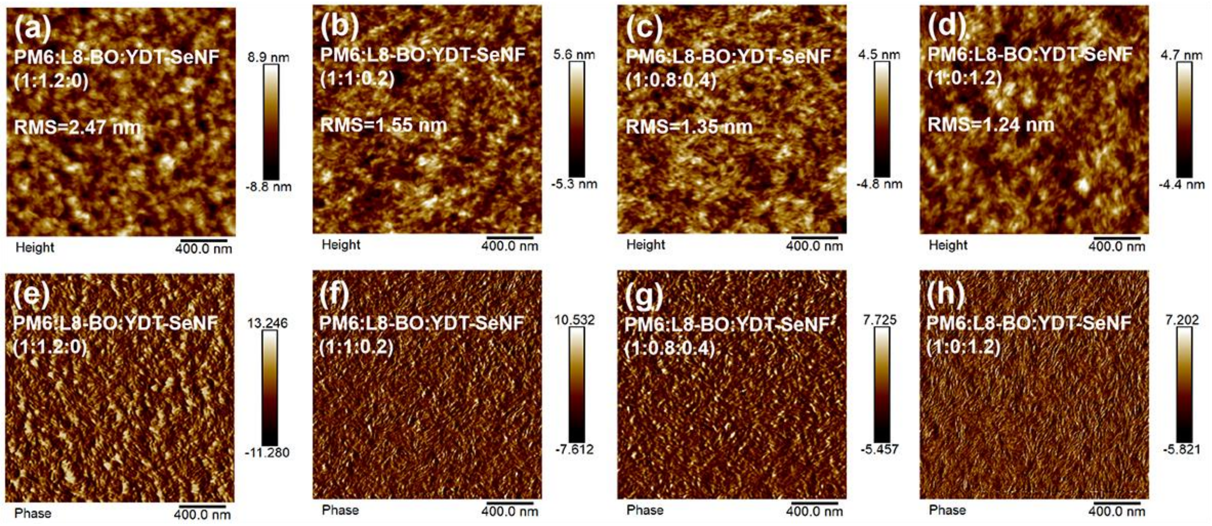


**Fig. S28** AFM height images (**a-d**) and phase images (**e-h**) of PM6:L8-BO:YDT-SeNF blends with different component ratios

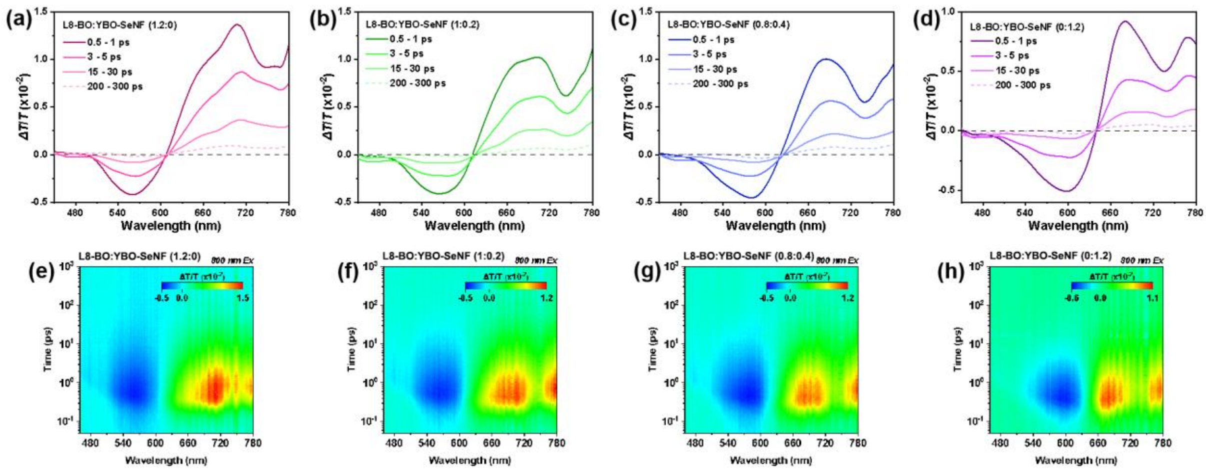


**Fig. S29** (**a-d**) Spectral line-cuts in terms of Δ*T/T* at representative times and (**e-h**) fs-TAS profiles of L8-BO:YBO-SeNF films with different component ratios

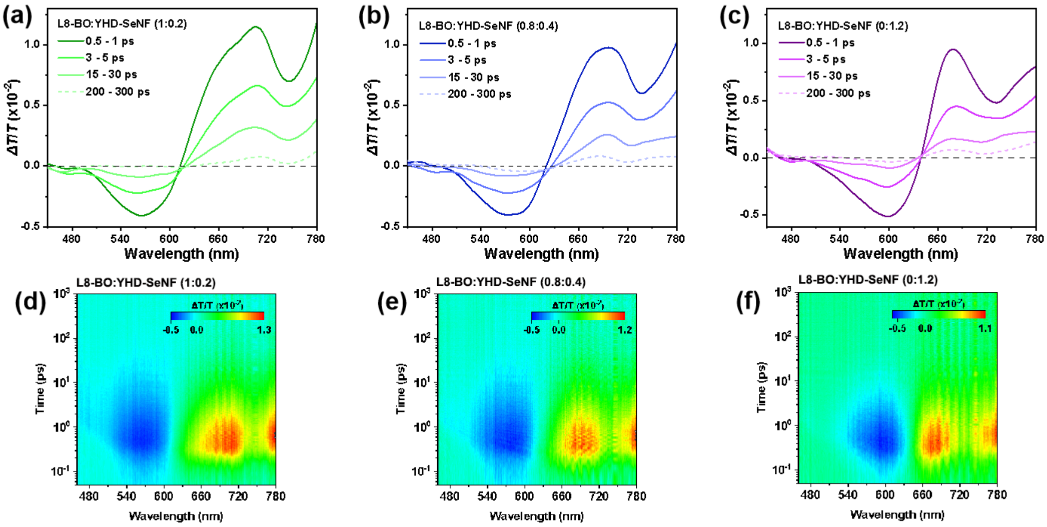


**Fig. S30** (**a-c**) Spectral line-cuts in terms of Δ*T/T* at representative times and (**d-f**) fs-TAS profiles of L8-BO:YHD-SeNF films with different component ratios

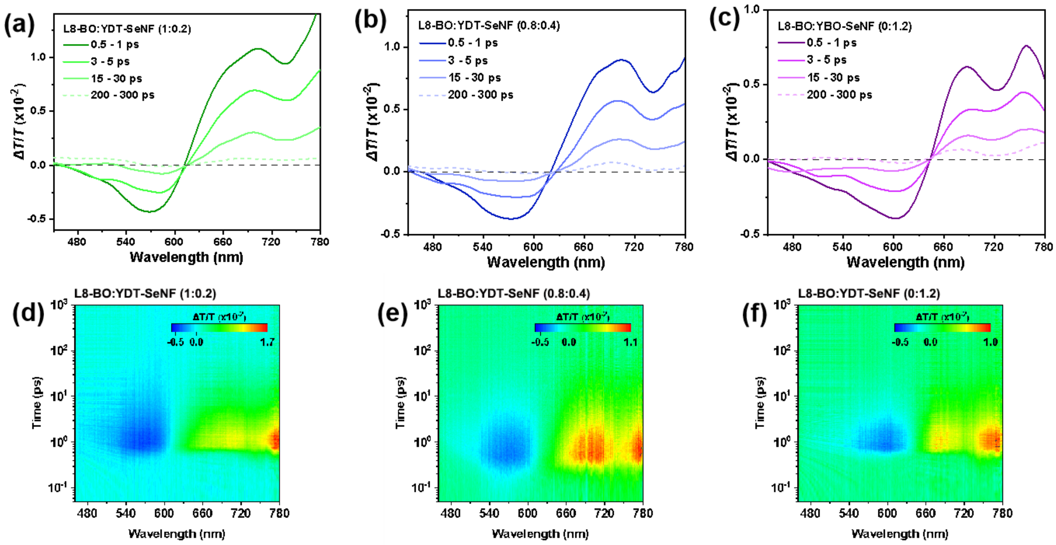


**Fig. S31** (**a-c**) Spectral line-cuts in terms of Δ*T/T* at representative times and (**d-f**) fs-TAS profiles of L8-BO:YDT-SeNF films with different component ratios

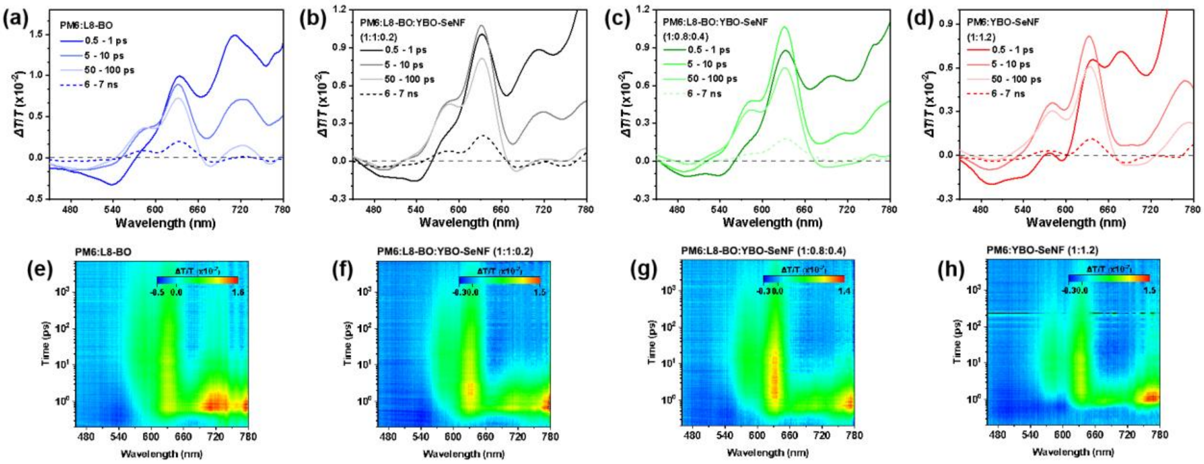


**Fig. S32** (**a-d**) Spectral line-cuts in terms of Δ*T/T* at representative times and (**e-h**) fs-TAS profiles of PM6:L8-BO:YBO-SeNF blend films with different component ratios

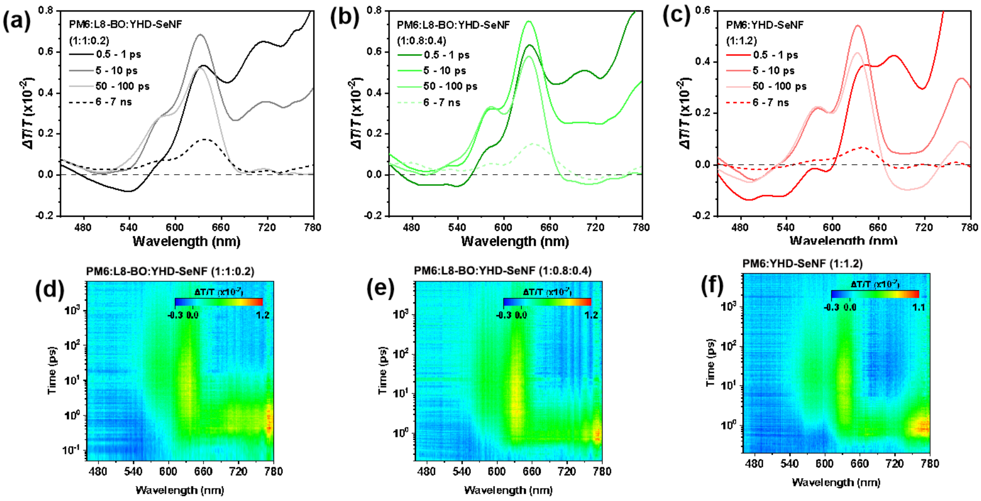


**Fig. S33** (**a-c**) Spectral line-cuts in terms of Δ*T*/*T* at representative times and (**d-f**) fs-TAS profiles of PM6:L8-BO:YHD-SeNF blend films with different component ratios

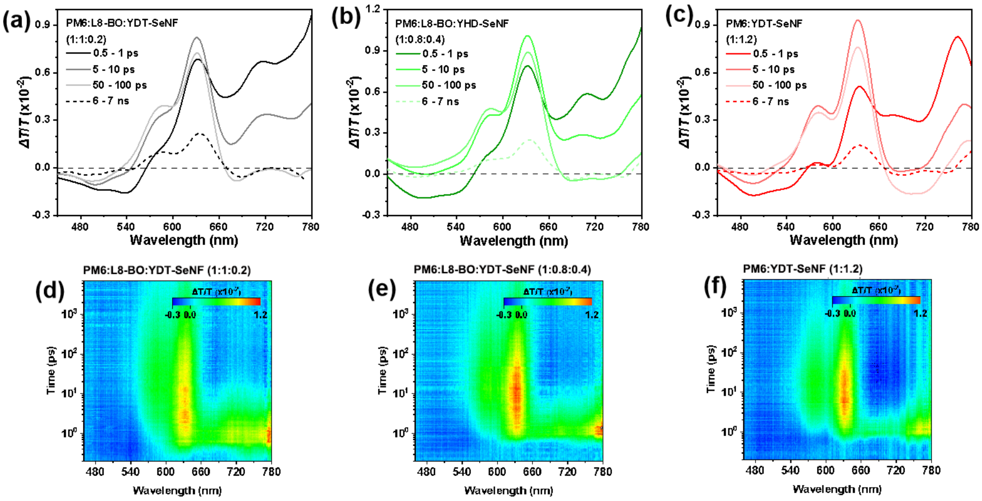


**Fig. S34** (**a-c**) Spectral line-cuts in terms of Δ*T*/*T* at representative times and (**d-f**) fs-TAS profiles of PM6:L8-BO:YDT-SeNF blend films with different component ratios

## Supplementary References

[S1] Y. Cui, H. Yao, J. Zhang, K. Xian, T. Zhang et al., Single-Junction Organic Photovoltaic Cells with Approaching 18% Efficiency. Adv. Mater. 32, 1908205 (2020). https://doi.org/10.1002/adma.201908205

[S2] F. Lin, K. Jiang, W. Kaminsky, Z. Zhu, A. K. Y. Jen, A Non-fullerene Acceptor with Enhanced Intermolecular π-Core Interaction for High-Performance Organic Solar Cells. J. Am. Chem. Soc. 142, 15246-15251 (2020). https://doi.org/10.1021/jacs.0c07083

[S3] Q. Fan, R. Ma, W. Su, Q. Zhu, Z. Luo et al., A new perspective to develop regiorandom polymer acceptors with high active layer ductility, excellent device stability, and high efficiency approaching 17%. Carbon Energy 5, e267 (2023). https://doi.org/10.1002/cey2.267

[S4] C. Li, J. Zhou, J. Song, J. Xu, H. Zhang et al., Non-fullerene acceptors with branched side chains and improved molecular packing to exceed 18% efficiency in organic solar cells. Nat. Energy 6, 605-613 (2021). https://doi.org/10.1038/s41560-021-00820-x

[S5] M. Zhang, X. Guo, W. Ma, H. Ade, J. Hou, A Large-Bandgap Conjugated Polymer for Versatile Photovoltaic Applications with High Performance. Adv. Mater. 27, 4655-4660 (2015). https://doi.org/10.1002/adma.201502110

[S6] Y. Shi, J. Pan, J. Yu, J. Zhang, F. Gao et al., Optimizing the Charge Carrier and Light Management of Nonfullerene Acceptors for Efficient Organic Solar Cells with Small Nonradiative Energy Losses. Sol. RRL 5, 2100008 (2021). https://doi.org/10.1002/solr.202100008

[S7] J. Yin, K. Chaitanya, X.-H. Ju, Bromination and cyanation for improving electron transport performance of anthra-tetrathiophene. J. Mater. Res. 31, 337-347 (2016). https://doi.org/10.1557/jmr.2016.8

[S8] A. Wadsworth, R. S. Ashraf, M. Abdelsamie, S. Pont, M. Little et al., Highly Efficient and Reproducible Nonfullerene Solar Cells from Hydrocarbon Solvents. ACS Energy Lett. 2, 1494-1500 (2017). https://doi.org/10.1021/acsenergylett.7b00390

[S9] Z. Zheng, O. M. Awartani, B. Gautam, D. Liu, Y. Qin et al., Efficient Charge Transfer and Fine-Tuned Energy Level Alignment in a THF-Processed Fullerene-Free Organic Solar Cell with 11.3% Efficiency. Adv. Mater. 29, 1604241 (2017). https://doi.org/10.1002/adma.201604241

[S10] L. Ye, Y. Xiong, Q. Zhang, S. Li, C. Wang et al., Surpassing 10% Efficiency Benchmark for Nonfullerene Organic Solar Cells by Scalable Coating in Air from Single Nonhalogenated Solvent. Adv. Mater. 30, 1705485 (2018). https://doi.org/10.1002/adma.201705485

[S11] X. Xu, T. Yu, Z. Bi, W. Ma, Y. Li, Q. Peng, Realizing Over 13% Efficiency in Green-Solvent-Processed Nonfullerene Organic Solar Cells Enabled by 1,3,4-Thiadiazole-Based Wide-Bandgap Copolymers. Adv. Mater. 30, 1703973 (2018) https://doi.org/10.1002/adma.201703973

[S12] W. Zhao, S. Zhang, Y. Zhang, S. Li, X. Liu et al., Environmentally Friendly Solvent-Processed Organic Solar Cells that are Highly Efficient and Adaptable for the Blade-Coating Method. Adv. Mater. 30, 1704837 (2018). https://doi.org/10.1002/adma.201704837

[S13] Y. Qin, L. Ye, S. Zhang, J. Zhu, B. Yang et al., A polymer design strategy toward green solvent processed efficient non-fullerene polymer solar cells. J Mater. Chem. A 6, 4324-4330 (2018). https://doi.org/10.1039/C8TA00368H

[S14] Q. Fan, Q. Zhu, Z. Xu, W. Su, J. Chen et al., Chlorine substituted 2D-conjugated polymer for high-performance polymer solar cells with 13.1% efficiency via toluene processing. Nano Energy 48, 413-420 (2018). https://doi.org/10.1016/j.nanoen.2018.04.002

[S15] X. Cheng, M. Li, Z. Guo, J. Yu, G. Lu et al., "Twisted" conjugated molecules as donor materials for efficient all-small-molecule organic solar cells processed with tetrahydrofuran. J. Mater. Chem. A 7, 23008-23018 (2019). https://doi.org/10.1039/C9TA07760J

[S16] U. K. Aryal, S. S. Reddy, K. Kranthiraja, J. Kim, W. Cho et al., Nonhalogenated Solvent-Processed Fullerene-Free Ambient Stable Organic Solar Cells: Impact of Molecular Weight of New π-Conjugated Donor Polymer on Efficiency. ACS Appl. Energy Mater. 2, 4159-4166 (2019). https://doi.org/10.1021/acsaem.9b00365

[S17] C. Liao, M. Zhang, X. Xu, F. Liu, Y. Li et al., Green solvent-processed efficient non-fullerene organic solar cells enabled by low-bandgap copolymer donors with

EDOT side chains. J. Mater. Chem. A 7, 716-726 (2019). https://doi.org/10.1039/C8TA10882J

[S18] Q. Liao, K. Yang, J. Chen, C. W. Koh, Y. Tang et al., (2019). Backbone Coplanarity Tuning of 1,4-Di(3-alkoxy-2-thienyl)-2,5-difluorophenylene-Based Wide Bandgap Polymers for Efficient Organic Solar Cells Processed from Nonhalogenated Solvent. ACS Appl. Mater. Interfaces 11, 31119-31128. https://doi.org/10.1021/acsami.9b09692

[S19] Y. Tang, H. Sun, Z. Wu, Y. Zhang, G. Zhang et al., A New Wide Bandgap Donor Polymer for Efficient Nonfullerene Organic Solar Cells with a Large Open-Circuit Voltage. Adv. Sci. 6, 1901773 (2019). https://doi.org/10.1002/advs.201901773

[S20] S. Dong, K. Zhang, B. Xie, J. Xiao, H.-L. Yip et al., High-Performance Large-Area Organic Solar Cells Enabled by Sequential Bilayer Processing via Nonhalogenated Solvents. Adv. Energy Mater. 9, 1802832 (2019). https://doi.org/10.1002/aenm.201802832

[S21] S. J. Jeon, Y. W. Han, D. K. Moon, 13.9%-Efficiency and Eco-Friendly Nonfullerene Polymer Solar Cells Obtained by Balancing Molecular Weight and Solubility in Chlorinated Thiophene-Based Polymer Backbones. Small 15, 1902598 (2019). https://doi.org/10.1002/smll.201902598

[S22] J. Chen, G. Li, Q. Zhu, X. Guo, Q. Fan et al., Highly efficient near-infrared and semitransparent polymer solar cells based on an ultra-narrow bandgap nonfullerene acceptor. J. Mater. Chem. A 7, 3745-3751 (2019). https://doi.org/10.1039/C8TA11484F

[S23] Y. Cui, H. Yao, L. Hong, T. Zhang, Y. Xu, et al., Achieving Over 15% Efficiency in Organic Photovoltaic Cells via Copolymer Design. Adv. Mater. 31, 1808356 (2019). https://doi.org/10.1002/adma.201808356

[S24] L. Hong, H. Yao, Z. Wu, Y. Cui, T. Zhang et al., Eco-Compatible Solvent-Processed Organic Photovoltaic Cells with Over 16% Efficiency. Adv. Mater. 31, 1903441 (2019). https://doi.org/10.1002/adma.201903441

[S25] R. Sun, T. Wang, Z. Luo, Z. Hu, F. Huang et al. Achieving Eco-Compatible Organic Solar Cells with Efficiency >16.5% Based on an Iridium Complex-Incorporated Polymer Donor. Sol. RRL 4, 2000156 (2020). https://doi.org/10.1002/solr.202000156

[S26] S. Dong, T. Jia, K. Zhang, J. Jing, F. Huang, Single-Component Non-halogen Solvent-Processed High-Performance Organic Solar Cell Module with Efficiency over 14%. Joule 4, 2004-2016 (2020). https://doi.org/10.1016/j.joule.2020.07.028

[S27] H. Huang, X. Li, C. Sun, I. Angunawela, B. Qiu et al., Green solvent-processed organic solar cells based on a low cost polymer donor and a small molecule acceptor. J. Mater. Chem. C 8, 7718-7724 (2020). https://doi.org/10.1039/D0TC01313G

[S28] C.-H. Zhang, F. Lin, W. Huang, J. Xin, J. Wang et al., Methyl functionalization on conjugated side chains for polymer solar cells processed from non-chlorinated solvents. J. Mater. Chem. C 8, 11532-11539 (2020). https://doi.org/10.1039/D0TC02032J

[S29] X. Xu, L. Yu, H. Yan, R. Li, Q. Peng, Highly efficient non-fullerene organic solar cells enabled by a delayed processing method using a non-halogenated solvent. Energy Environ. Sci. 13, 4381-4388 (2020). https://doi.org/10.1039/D0EE02034F

[S30] B. Fan, F. Lin, J. Oh, H. Fu, W. Gao et al., Enabling High Efficiency of Hydrocarbon-Solvent Processed Organic Solar Cells through Balanced Charge

Generation and Non-Radiative Loss. Adv. Energy Mater. 11, 2101768 (2021). https://doi.org/10.1002/aenm.202101768

[S31] C. Kim, S. Chen, J. S. Park, G.-U. Kim, H. Kang et al., Green solvent-processed, high-performance organic solar cells achieved by outer side-chain selection of selenophene-incorporated Y-series acceptors. J. Mater. Chem. A 9, 24622-24630 (2021). https://doi.org/10.1039/D1TA07046K

[S32] H. Chen, H. Lai, Z. Chen, Y. Zhu, H. Wang et al., 17.1 %-Efficient Eco-Compatible Organic Solar Cells from a Dissymmetric 3D Network Acceptor. Angew. Chem. Int. Ed. 60, 3238-3246 (2021). https://doi.org/10.1002/anie.202013053

[S33] S. Qin, Z. Jia, L. Meng, C. Zhu, W. Lai et al., Non-Halogenated-Solvent Processed and Additive-Free Tandem Organic Solar Cell with Efficiency Reaching 16.67%. Adv. Funct. Mater. 31, 2102361 (2021). https://doi.org/10.1002/adfm.202102361

[S34] W. Sun, H. Chen, B. Zhang, Q. Cheng, H. Yang et al., Host-Guest Active Layer Enabling Annealing-Free, Nonhalogenated Green Solvent Processing for High-Performance Organic Solar Cells. Chin. J. Chem. 40, 2963-2972 (2022). https://doi.org/10.1002/cjoc.202200437

[S35] J. Wan, L. Zeng, X. Liao, Z. Chen, S. Liu et al., All-Green Solvent-Processed Planar Heterojunction Organic Solar Cells with Outstanding Power Conversion Efficiency of 16%. Adv. Funct. Mater. 32, 2107567 (2022). https://doi.org/10.1002/adfm.202107567

[S36] J. Zhang, Q. Huang, K. Zhang, T. Jia, J. Jing et al., Random copolymerization strategy for non-halogenated solvent-processed all-polymer solar cells with a high efficiency of over 17%. Energy Environ. Sci. 15, 4561-4571 (2022). https://doi.org/10.1039/D2EE01996E

[S37] K. Hu, C. Zhu, K. Ding, S. Qin, W. Lai et al., Solid additive tuning of polymer blend morphology enables non-halogenated-solvent all-polymer solar cells with an efficiency of over 17%. Energy Environ. Sci. 15, 4157-4166 (2022). https://doi.org/10.1039/D2EE01727J

[S38] J. Wan, Y. Wu, R. Sun, J. Qiao, X. Hao et al., An alloy small molecule acceptor for green printing organic solar cells overcoming the scaling lag of efficiency. Energy Environ. Sci. 15, 5192-5201 (2022). https://doi.org/10.1039/D2EE03134E

[S39] H. Xia, Y. Zhang, W. Deng, K. Liu, X. Xia et al. Novel Oligomer Enables Green Solvent Processed 17.5% Ternary Organic Solar Cells: Synergistic Energy Loss Reduction and Morphology Fine-Tuning. Adv. Mater. 34, 2107659 (2022). https://doi.org/10.1002/adma.202205638

[S40] H. Lu, H. Wang, G. Ran, S. Li, J. Zhang et al., Random Terpolymer Enabling High-Efficiency Organic Solar Cells Processed by Nonhalogenated Solvent with a Low Nonradiative Energy Loss. Adv. Funct. Mater. 32, 2203193 (2022). https://doi.org/10.1002/adfm.202203193

[S41] J. Liu, J. Deng, Y. Zhu, X. Geng, L. Zhang et al., Regulation of Polymer Configurations Enables Green Solvent-Processed Large-Area Binary All-Polymer Solar Cells With Breakthrough Performance and High Efficiency Stretchability Factor. Adv. Mater. 35, 2208008 (2023). https://doi.org/10.1002/adma.202208008

[S42] S. Pang, Z. Chen, J. Li, Y. Chen, Z. Liu et al., High-efficiency organic solar cells processed from a real green solvent. Mater. Horiz. 10, 473-482 (2023).

https://doi.org/10.1039/D2MH01314B

[S43] J. Shi, Z. Chen, Y. Qiu, M. Luo, X. Zhang et al., Facile Side Chains Optimization of Y-series Acceptor Enables High Performance Binary Non-halogenated Solvent-Processed Organic Solar Cells with Excellent Fill Factor of 79%. Sol. RRL 7, 2300206 (2023). https://doi.org/10.1002/solr.202300206

[S44] D. Vak, K. Hwang, A. Faulks, Y.-S. Jung, N. Clark et al., 3D Printer Based Slot-Die Coater as a Lab-to-Fab Translation Tool for Solution-Processed Solar Cells. Adv. Energy Mater. 5, 1401539 (2015). https://doi.org/10.1002/aenm.201401539

[S45] H. W. Ro, J. M. Downing, S. Engmann, A. A. Herzing, D. M. DeLongchamp et al., Morphology changes upon scaling a high-efficiency, solution-processed solar cell. Energy Environ. Sci. 9, 2835-2846 (2016). https://doi.org/10.1039/C6EE01623E

[S46] S. Hong, H. Kang, G. Kim, S. Lee, S. Kim et al., A series connection architecture for large-area organic photovoltaic modules with a 7.5% module efficiency. Nat. Commun. 7, 10279 (2016). https://doi.org/10.1038/ncomms10279

[S47] L. Zhang, X. Xu, B. Lin, H. Zhao, T. Li et al., (2018). Achieving Balanced Crystallinity of Donor and Acceptor by Combining Blade-Coating and Ternary Strategies in Organic Solar Cells. Adv. Mater. 30, 1805041. https://doi.org/10.1002/adma.201805041

[S48] S. Strohm, F. Machui, S. Langner, P. Kubis, N. Gasparini et al., P3HT: non-fullerene acceptor based large area, semi-transparent PV modules with power conversion efficiencies of 5%, processed by industrially scalable methods. Energy Environ. Sci. 11, 2225-2234 (2018). https://doi.org/10.1039/C8EE01150H

[S49] Q. Kang, L. Ye, B. Xu, C. An, S. J. Stuard et al., A Printable Organic Cathode Interlayer Enables over 13% Efficiency for 1-cm2 Organic Solar Cells. Joule 3, 227-239 (2019). https://doi.org/10.1016/j.joule.2018.10.024

[S50] S. Dong, K. Zhang, T. Jia, W. Zhong, X. Wang et al., Suppressing the excessive aggregation of nonfullerene acceptor in blade-coated active layer by using n-type polymer additive to achieve large-area printed organic solar cells with efficiency over 15%. EcoMat 1, e12006 (2019). https://doi.org/10.1002/eom2.12006

[S51] G. Ji, W. Zhao, J. Wei, L. Yan, Y. Han et al., 12.88% efficiency in doctor-blade coated organic solar cells through optimizing the surface morphology of a ZnO cathode buffer layer. J. Mater. Chem. A 7, 212-220 (2019). https://doi.org/10.1039/C8TA08873J

[S52] L. Zhang, H. Zhao, B. Lin, J. Yuan, X. Xu et al., A blade-coated highly efficient thick active layer for non-fullerene organic solar cells. J. Mater. Chem. A 7, 22265-22273 (2019). https://doi.org/10.1039/C9TA09799F

[S53] Q. Kang, Q. Liao, Y. Xu, L. Xu, Y. Zu et al., p-Doped Conducting Polyelectrolyte as an Anode Interlayer Enables High Efficiency for 1 cm2 Printed Organic Solar Cells. ACS Appl. Mater. Interfaces 11, 20205-20213 (2019). https://doi.org/10.1021/acsami.9b04211

[S54] S. Rasool, D. V. Vu, C. E. Song, H. K. Lee, S. K. Lee et al., Room Temperature Processed Highly Efficient Large-Area Polymer Solar Cells Achieved with Molecular Engineering of Copolymers. Adv. Energy Mater. 9, 1900168 (2019). https://doi.org/10.1002/aenm.201900168

[S55] S.-H. Chen, C. H. Liao, C. Y. Chang, K. M. Huang, J. Y. Chen et al. Large-area blade-coated organic solar cells processed from halogen-free solvent. Org. Electron.

75, 105376 (2019). https://doi.org/10.1016/j.orgel.2019.105376

[S56] R. Sun, J. Guo, C. Sun, T. Wang, Z. Luo et al. A universal layer-by-layer solution-processing approach for efficient non-fullerene organic solar cells. Energy Environ. Sci. 12, 384-395 (2019). https://doi.org/10.1039/C8EE02560F

[S57] Q. Wu, J. Guo, R. Sun, J. Guo, S. Jia et al., Slot-die printed non-fullerene organic solar cells with the highest efficiency of 12.9% for low-cost PV-driven water splitting. Nano Energy 61, 559-566 (2019). https://doi.org/10.1016/j.nanoen.2019.04.091

[S58] R. Sun, J. Guo, Q. Wu, Z. Zhang, W. Yang et al., A multi-objective optimization-based layer-by-layer blade-coating approach for organic solar cells: rational control of vertical stratification for high performance. Energy Environ. Sci. 12, 3118-313 (2019). https://doi.org/10.1039/C9EE02295C

[S59] C. Zhu, H. Huang, Z. Jia, F. Cai, J. Li et al., Spin-coated 10.46% and blade-coated 9.52% of ternary semitransparent organic solar cells with 26.56% average visible transmittance. Sol. Energy 204, 660-666 (2020). https://doi.org/10.1016/j.solener.2020.05.027

[S60] X. Han, J. Zhu, Y. Xiao, H. Jiang, Z. Zhang et al., An Alkoxy-Solubilizing Decacyclic Electron Acceptor for Efficient Ecofriendly As-Cast Blade-Coated Organic Solar Cells. Sol. RRL 4, 2000108 (2020). https://doi.org/10.1002/solr.202000108

[S61] Y. Cui, H. Yao, L. Hong, T. Zhang, Y. Tang et al., Organic photovoltaic cell with 17% efficiency and superior processability. Natl. Sci. Rev. 7, 1239-1246 (2020). https://doi.org/10.1093/nsr/nwz200

[S62] K. Weng, L. Ye, L. Zhu, J. Xu, J. Zhou et al., Optimized active layer morphology toward efficient and polymer batch insensitive organic solar cells. Nat. Commun. 11, 2855 (2020). https://doi.org/10.1038/s41467-020-16621-x

[S63] R. Sun, Q. Wu, J. Guo, T. Wang, Y. Wu et al. A Layer-by-Layer Architecture for Printable Organic Solar Cells Overcoming the Scaling Lag of Module Efficiency. Joule 4, 407-419 (2020). https://doi.org/10.1016/j.joule.2019.12.004

[S64] Y. Wang, X. Wang, B. Lin, Z. Bi, X. Zhou et al., Achieving Balanced Crystallization Kinetics of Donor and Acceptor by Sequential-Blade Coated Double Bulk Heterojunction Organic Solar Cells. Adv. Energy Mater. 10, 2000826 (2020). https://doi.org/10.1002/aenm.202000826

[S65] Y. Li, H. Liu, J. Wu, H. Tang, H. Wang et al., Additive and High-Temperature Processing Boost the Photovoltaic Performance of Nonfullerene Organic Solar Cells Fabricated with Blade Coating and Nonhalogenated Solvents. ACS Appl. Mater. Interfaces 13, 10239-10248 (2021). https://doi.org/10.1021/acsami.0c23035

[S66] J. Yuan, D. Liu, H. Zhao, B. Lin, X. Zhou et al., Patterned Blade Coating Strategy Enables the Enhanced Device Reproducibility and Optimized Morphology of Organic Solar Cells. Adv. Energy Mater. 11, 2100098 (2021). https://doi.org/10.1002/aenm.202100098

[S67] Y. Zhang, K. Liu, J. Huang, X. Xia, J. Cao et al. Graded bulk-heterojunction enables 17% binary organic solar cells via nonhalogenated open air coating. Nat. Commun. 12, 4815 (2021). https://doi.org/10.1038/s41467-021-25148-8

[S68] H. Zhao, B. Lin, J. Xue, H. B. Naveed, C. Zhao et al., (2022). Kinetics Manipulation Enables High-Performance Thick Ternary Organic Solar Cells via R2R-Compatible Slot-Die Coating. Adv. Mater. 34, 2105114. https://doi.org/10.1002/adma.202105114

[S69] Q. Zhu, J. Xue, G. Lu, B. Lin, H. B. Naveed et al., Efficient and mechanically-robust organic solar cells based on vertical stratification modulation through sequential blade-coating. Nano Energy 97, 107194 (2022). https://doi.org/10.1016/j.nanoen.2022.107194

[S70] J. Xue, H. Zhao, B. Lin, Y. Wang, Q. Zhu et al., Nonhalogenated Dual-Slot-Die Processing Enables High-Efficiency Organic Solar Cells. Adv. Mater. 34, 2202659 (2022). https://doi.org/10.1002/adma.202202659

[S71] S R. Sun, T. Wang, X. Yang, Y. Wu, Y. Wang et al., High-speed sequential deposition of photoactive layers for organic solar cell manufacturing. Nat. Energy 7, 1087-1099 (2022). https://doi.org/10.1038/s41560-022-01140-4
